# How Accurate Are Low-Cost Air Quality Sensors? A 10-Month Study of Three-Tier Comparison with Three Calibration Methods and Cross-Site Transferability

**DOI:** 10.3390/s26206441

**Published:** 2026-10-12

**Authors:** Andres Aharhel Mercado-Velazquez, Ponciano Jorge Escamilla-Ambrosio, Eliane Bodanese, Muhammad Islam, Fesal Toosy, Gioia Mosler, Stefan Poslad

**Affiliations:** 1 School of Electronic Engineering and Computer Science, Queen Mary University of London, London E1 4NS, UK; a.mercadovelazquez@qmul.ac.uk (A.A.M.-V.); eliane.bodanese@qmul.ac.uk (E.B.); stefan.poslad@qmul.ac.uk (S.P.); 2 Centro de Investigación en Computación, Instituto Politécnico Nacional, Av. Juan de Dios Bátiz esq. Miguel Othón de Mendizábal, Col. Nueva Industrial Vallejo, Alcaldía Gustavo A. Madero, Mexico City 07738, Mexico; 3 Pollution Team, London Borough of Tower Hamlets, London E1 1BJ, UK; muhammad.islam@towerhamlets.gov.uk; 4 Department of Electrical Engineering, University of Central Punjab, 1 Khayaban-e-Jinnah, Johar Town, Lahore 54782, Pakistan; fesal@ucp.edu.pk; 5 Centre for Genomics and Child Health, Blizard Institute, Barts and the London School of Medicine and Dentistry, Queen Mary University of London, 4 Newark Street, London E1 2AT, UK; g.mosler@qmul.ac.uk

**Keywords:** air quality monitoring, cross-site transferability, electrochemical sensors, low-cost sensors, machine learning, metal oxide semiconductor gas sensors, nitrogen dioxide, particulate matter, sensor calibration, sensor drift

## Abstract

**Highlights:**

**What are the main findings?**

- With machine-learning calibration, low-cost sensors reach $R^{2}=0.58$–0.74 for ${\mathrm{PM}}_{2.5}$ and 0.23–0.37 for ${\mathrm{NO}}_{2}$ against government reference stations, and 0.58–0.79 for CO against a factory-calibrated mid-cost electrochemical sensor, across the two sites on which this study focuses.
- ${\mathrm{PM}}_{2.5}$ calibration transfers between sites with limited loss ($|\Delta R^{2}|$≤0.18). The ${\mathrm{NO}}_{2}$ calibration trained on April–October 2025 data stops describing the November 2025–February 2026 data at both sites, and the CO calibration at one.

**What are the implications of the main findings?**

- Calibrated at a reference site, low-cost sensors could deliver ${\mathrm{PM}}_{2.5}$ data at the neighborhood scale where reference stations are not available. ${\mathrm{NO}}_{2}$ requires a co-location at each site.
- For ${\mathrm{PM}}_{2.5}$, a linear calibration with temperature and relative humidity equals machine learning and transfers between sites. Machine learning outperforms it for the metal oxide CO sensor at both sites and for ${\mathrm{NO}}_{2}$ at one.

**Abstract:**

Low-cost sensors could increase the spatial granularity of air-quality monitoring at 700–1000 times less cost than reference stations (2026 prices), but few field studies evaluate them for gases. This study evaluates a three-tier architecture (low-cost metal oxide and optical, mid-cost electrochemical, high-cost government reference) over 10 months at two London sites, 1.6 km apart. The low-cost sensors and mid-cost ${\mathrm{NO}}_{2}$ sensor were calibrated with manufacturer curves, linear regression on temperature and relative humidity, and machine learning (ML). After field calibration, the low-cost ${\mathrm{PM}}_{2.5}$ sensor explains over half the reference variance at both sites, and linear calibration equals ML. The low-cost ${\mathrm{NO}}_{2}$ sensor agrees less with its reference than the ${\mathrm{PM}}_{2.5}$ sensor. The low-cost sensors explain over half the variance of a factory-calibrated mid-cost electrochemical CO sensor at both sites only with ML. The mid-cost ${\mathrm{NO}}_{2}$ and low-cost O_3_ sensors fail under all three methods. Calibrations trained on April–October 2025 stop describing November 2025–February 2026 data for ${\mathrm{NO}}_{2}$ at both sites and CO at one, whereas ${\mathrm{PM}}_{2.5}$ stays stable. Transferred between sites, ${\mathrm{PM}}_{2.5}$ calibrations reach $R^{2}\geq 0.56$ and gas calibrations $R^{2}\leq 0.28$. Low-cost ${\mathrm{PM}}_{2.5}$ sensors calibrated at a reference site could serve neighborhood-scale monitoring where reference stations are absent, whereas ${\mathrm{NO}}_{2}$ needs co-location.

## 1. Introduction

### 1.1. The Spatial Granularity Problem

An estimated 7.9 million deaths in 2023 were attributable to air pollution, of which approximately 86% resulted from noncommunicable diseases such as heart disease, chronic obstructive pulmonary disease (COPD), diabetes, and dementia [1]. In 2021, the World Health Organization (WHO) updated its global air quality guidelines (AQG) for six priority pollutants, lowering most guideline levels: particulate matter (${\mathrm{PM}}_{2.5}$ and ${\mathrm{PM}}_{10}$, with aerodynamic diameters of ≤2.5 μm and ≤10 μm), nitrogen dioxide (${\mathrm{NO}}_{2}$), ozone (O_3_), sulfur dioxide (${\mathrm{SO}}_{2}$), and carbon monoxide (CO), following epidemiological evidence of adverse health effects at concentrations below previous thresholds [2]. Accurate exposure assessment therefore requires monitoring at the neighborhood scale where people live, work, and commute.

Each street, intersection, and building facade creates a distinct microclimate where primary traffic-related pollutants such as nitric oxide (NO), ${\mathrm{NO}}_{2}$, and black carbon can vary by more than five-fold within individual city blocks and by more than eight-fold across the broader urban area [3]. Despite this fine-scale variability, reference monitoring networks remain sparse. London’s Air Quality Network (LAQN), one of the world’s densest urban reference networks, managed by the Environmental Research Group at Imperial College London, operates over 100 continuous reference-grade monitoring stations (chemiluminescence analyzers for ${\mathrm{NO}}_{2}$, Beta Attenuation Monitors (BAM) for ${\mathrm{PM}}_{2.5}$ and ${\mathrm{PM}}_{10}$, UV photometric analyzers for O_3_) across Greater London’s 1572 km^2^ [4,5]. This corresponds to one station per 16 km^2^ (adjacent stations roughly 4 km apart), far coarser than the street and block scales at which these pollutants vary [3]; a single station therefore cannot resolve variations within its own coverage area. Outside these zones, exposure assessments rely on spatial interpolation or dispersion modeling [6], both uncertain at the neighborhood level, which limits the evidence base for targeted public-health interventions and enforcing air quality standards such as EU Directive 2024/2881 [7] (recast of Directive 2008/50/EC, applicable from December 2026).

The primary barrier to increasing spatial granularity is cost. A single such reference-grade station costs approximately $27,000 USD for capital equipment alone, an estimate provided by the Pollution Team of the London Borough of Tower Hamlets in April 2026. Annual expenses for service and maintenance, data management, and quality assurance/quality control (QA/QC) add to this figure [8]. Deploying hundreds of such stations to achieve neighborhood-scale coverage is economically prohibitive for all but the most well-funded metropolitan authorities. Throughout this paper, the cost ratios (1×, ∼5–7×, ∼700–1000×) refer to per-sensor comparisons (Section 2.1). A fully assembled low-cost monitoring node, which adds single-board computer, housing, power, and connectivity to the sensors themselves, totals under $200 USD (April 2026), less than 1% of the reference-station capital cost. Component prices have risen since the network was assembled: several of the sensors used here were purchased in 2024–2025 at lower prices than the April 2026 figures quoted throughout.

Low-cost sensors offer a potential solution. Metal oxide gas sensors (e.g., GM-702B for CO, GM-102B for ${\mathrm{NO}}_{2}$, MQ131 for O_3_) and optical particle counters (e.g., Sensirion SPS30) are available at approximately 1/1000 the cost of a reference station per sensor, allowing hundreds of measurement points for the budget of one reference station. However, these metal oxide sensors (MEMS (micro-electro-mechanical systems) or conventional ceramic substrates) have received minimal published validation for ambient air quality applications. The relevant question is, therefore, not whether they are as accurate as reference instruments (they are not), but whether they are sufficiently accurate to provide useful information in locations where no other data exists.

This paper evaluates low-cost sensors for four criteria pollutants regulated by WHO AQG 2021 [2], EU Directive 2024/2881 [7], and the UK Air Quality Standards Regulations 2010 [9]: ${\mathrm{PM}}_{2.5}$, ${\mathrm{NO}}_{2}$, O_3_, and CO, which, together with other ambient pollutants, drive most of the urban disease burden associated with air pollution [1,2]. ${\mathrm{PM}}_{10}$ (co-measured by the optical particle counter) and total volatile organic compounds (VOC, measured by the SGP40 sensor) are retained as auxiliary input features for the machine learning models (Section 2.6); neither is evaluated as a calibration target because no co-located reference instrument is available.

### 1.2. State of the Art and Research Gaps

Evaluation of commercial air quality sensor systems has advanced substantially in recent years. Diez et al. [10] evaluated 43 sensor devices across three UK cities over three years in the QUANT study. Malings et al. [11] evaluated 68 RAMP monitors over deployments spanning up to 18 months for CO, ${\mathrm{NO}}_{2}$, O_3_, and other gases. Hayward et al. [12] analyzed over 470,000 correction combinations for AQMesh and other commercial systems at two UK sites, concluding that “results are more impacted by device type and deployment location than by the regression technique used” and that simple linear models perform comparably to complex non-parametric methods for electrochemical and optical sensors.

However, these studies focused predominantly on mid-cost electrochemical sensors (approximately 5–7 times the per-sensor cost of low-cost metal oxide semiconductor (MOS) sensors) and higher-cost multi-sensor modules. Field evaluations of individual MOS sensors at the low-cost tier are scarce. Peterson et al. [13] evaluated SGX Sensortech MICS-4514 and MICS-2614 sensors within the SOGS research test bed for ${\mathrm{NO}}_{2}$ and O_3_; Cavaliere et al. [14] evaluated SGX MiCS-2714 and MiCS-2614 sensors within the AirQino station in Italy. Neither study assessed the Winsen GM-702B (CO) or GM-102B (${\mathrm{NO}}_{2}$) micro-electro-mechanical systems (MEMS) sensors used here. For the MQ131 (O_3_), only Badura et al. [15] have reported a systematic field evaluation against co-located reference instruments.

Five specific gaps motivate this work:

**Gap 1 (Three-tier cost comparison):** At the time of writing, no study has systematically compared three cost tiers (low-cost metal oxide and optical as the cost baseline, mid-cost electrochemical at approximately 5–7 times that cost, and high-cost government reference at approximately 700–1000 times that cost) using the same calibration methods on identical co-located data. Existing comparisons evaluate two tiers at most: deSouza et al. [16] compared low-cost sensors against reference, and Vajs et al. [17] propagated ML calibration within a single-tier network. Without a three-tier comparison at the same site, the cost-performance trade-off for deployment decisions cannot be quantified directly.

**Gap 2 (Sensor evaluation):** The GM-102B (${\mathrm{NO}}_{2}$) and GM-702B (CO), both MEMS metal oxide sensors manufactured by Winsen and integrated into the Seeedstudio Grove Multichannel Gas Sensor v2, currently have no field evaluations published. The MQ131 (O_3_), a conventional metal oxide semiconductor sensor [15,18], has only one systematic field evaluation against co-located reference instruments: Badura et al. assessed three MQ131 units over 18 months in Wrocław, Poland, and report their agreement with the reference as the coefficient of determination $R^{2}$ ($R^{2}=1$ indicates perfect agreement with the reference, $R^{2}=0$ no linear correlation, and $R^{2}<0$ predictions worse than the reference mean). The MQ131 reached $R^{2}>0.86$ when its calibration included temperature and relative humidity, and $R^{2}=0.4$–0.7 when it did not. It also showed the largest inter-unit variability of the four sensor models tested, two low-cost and two medium-cost. The co-tested SensoriC O_3_ 3E 1 electrochemical sensor degraded from $R^{2}>0.90$ in the first 9 months to $R^{2}=0.83$ at month 13, reaching $R^{2}<0.04$ by study end due to electrolyte aging [15]. The gap is, therefore, the absence of any independent field validation for these specific MEMS MOS sensors at the low-cost tier.

**Gap 3 (Monthly diagnosis):** Aggregate metrics over entire deployments [10,11] or broad sub-periods [15] cannot resolve seasonal or progressive drift. At the time of writing, no study provides month-by-month $R^{2}$ series for individual metal oxide sensors across multiple gases over periods exceeding six months.

**Gap 4 (Cross-site transfer for metal oxide):** Cross-site calibration transferability has been evaluated for electrochemical and higher-cost semiconductor sensors but not, at the time of writing, for low-cost metal oxide devices. Abu-Hani et al. [19] reported ∼28% $R^{2}$ degradation when transferring machine learning (ML) models for Alphasense electrochemical ${\mathrm{NO}}_{2}$ sensors between sites. Winter et al. [20] achieved $R^{2}=0.88$ for remotely calibrated Alphasense O_3_ sensors at 17.5–29.1 km in the BEA${\mathrm{CO}}_{2}$N network. Miskell et al. [21] proposed hierarchical proxy calibration for semiconductor O_3_ sensors, with mean absolute error (MAE) of 3.0–5.6 ppb at proxy distances of 4.4–56.7 km. None of these studies evaluated metal oxide sensors at the low-cost tier.

**Gap 5 (OLS vs ML for metal oxide):** Hayward et al. [12] concluded that ordinary least squares (OLS) regression suffices for electrochemical and optical sensors. Whether this extends to metal oxide sensors, whose signal-concentration relationships are fundamentally non-linear, remains unevaluated.

### 1.3. Contributions

The main contributions of this paper are the following:

1. Quantification of the performance trade-off at each cost tier (low-cost MOS and optical as the cost baseline, mid-cost electrochemical at approximately 5–7 times that cost, and high-cost government reference at approximately 700–1000 times that cost), using three calibration methods on identical data at two independent sites (Section 3.1 and Section 4.1).
2. The first field evaluation, at the time of writing, of GM-102B (${\mathrm{NO}}_{2}$) and GM-702B (CO) for ambient air quality monitoring, and the second independent evaluation of MQ131 (O_3_) [15], in a different geographical context with a dedicated preprocessing pipeline (Section 2.2, Section 2.3 and Section 3.1).
3. A formalized, reproducible method for dynamic $R_{0}$ (the sensor resistance in clean air, re-estimated daily rather than fixed once at deployment) baseline estimation that addresses the drift problem inherent to metal oxide sensors [22], using exponentially weighted moving average (EWMA) smoothing and nighttime percentile selection, applicable to any MOS sensor in environmental monitoring (Section 2.3).
4. Monthly diagnosis over 10 months, identifying which calibrations fitted on the training data (April to October 2025) stop describing the test data (November 2025 to February 2026) and which remain stable (Section 3.4).
5. The first quantification, at the time of writing, of cross-site calibration transfer for the GM-102B and GM-702B sensors, evaluated together with the SPS30 optical sensor in three scenarios that use increasing amounts of destination information (Section 2.7). Applied unchanged, the models trained by co-location at a government reference site extend to ${\mathrm{PM}}_{2.5}$ at a site without reference ($|\Delta R^{2}|$≤0.18), whereas ${\mathrm{NO}}_{2}$, CO and O_3_ do not transfer. For CO and O_3_ the models are trained against an individual factory-calibrated mid-cost electrochemical sensor rather than a standardized government instrument (Section 3.5 and Section 4.4).
6. Evidence, on the same test data, of where a linear calibration with temperature and relative humidity suffices and where ML is justified. The linear model suffices for the optical ${\mathrm{PM}}_{2.5}$ sensor, also over the test months and across the two sites, whereas ML is justified for the metal oxide CO sensor at both sites and for ${\mathrm{NO}}_{2}$ at one site only. This confirms the conclusion of Hayward et al. [12] that linear models suffice for the optical sensor, but not for the metal oxide CO sensor (Section 3.1 and Section 4.3).

The remainder of this paper is organized as follows. Section 2 describes the sensor network, preprocessing pipeline, three calibration methods, cross-node transfer protocol, and evaluation metrics. Section 3 presents results for the overall performance comparison, ML model selection, feature configurations (the model inputs), monthly degradation, and cross-node transferability. Section 4 discusses the cost-performance trade-off, the O_3_ failure, when ML is justified over a linear calibration, transferability for network deployment, and study limitations. Section 5 provides conclusions and recommendations.

## 2. Materials and Methods

### 2.1. Sensor Network and Three-Tier Architecture

**Figure 1:**
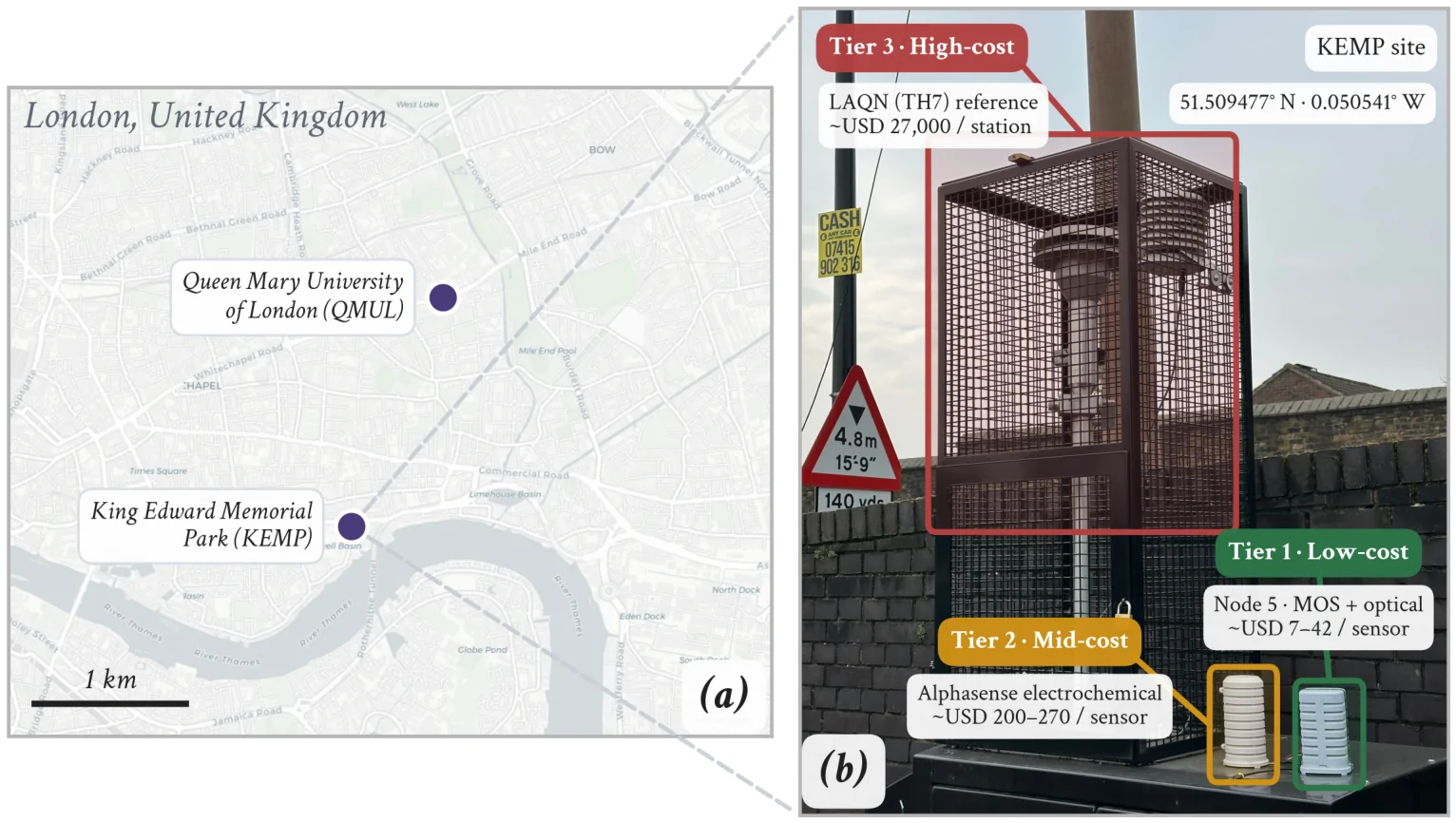
(**a**) Location of the two analyzed sites in east London, each co-located with a LAQN reference station (TH2 at QMUL, TH7 at KEMP). (**b**) In-situ three-tier co-location at KEMP (Node 5). Basemap: © OpenStreetMap contributors, © CARTO.

This study uses a low-cost air-quality monitoring network of seven nodes deployed across east London, whose node hardware, sensor selection rationale and data pipeline were described in a previous study [23]. It analyzes two of these nodes, Nodes 3 and 5, because they are the only ones where all three cost tiers are present. Each is co-located with a LAQN reference station, the high-cost tier (Figure 1, Table 1), and each carries mid-cost Alphasense electrochemical sensors next to its low-cost sensors, which no other node of the network has. Node 3 is at Queen Mary University of London (QMUL), on Mile End Road. Node 5 is at King Edward Memorial Park (KEMP), at the intersection of Glamis Road and the A1203 highway, approximately 1.6 km south of QMUL.

Each node is built around a Raspberry Pi Zero 2 W single-board computer [23]. The GM-702B and GM-102B (MEMS metal oxide sensors on silicon microheater substrates) come integrated in the Grove Multichannel Gas Sensor v2 carrier module; the MQ131, a conventional metal oxide semiconductor sensor with its sensing layer on a ceramic tube [15,18], is mounted on an FC-22 interface board. Table 2 lists the full sensor inventory.

Of the seven deployed nodes, only Nodes 3 and 5 (the two analyzed in this three-tier comparison study) carry Alphasense B-Series 4-electrode electrochemical sensors (Alphasense Ltd., Great Notley, Essex, UK), each mounted on an Individual Sensor Board (ISB). Costs in Table 2 include the ISB. The co-located reference stations TH2 and TH7 provide only ${\mathrm{NO}}_{2}$ and ${\mathrm{PM}}_{2.5}$ measurements at a sampling resolution of 15 min via the London Air API [24]. Neither station is equipped to measure CO, O_3_, or any other pollutants (Table 1). The Alphasense CO-B4 and OX-B431 therefore provide the concentrations against which the low-cost CO and O_3_ sensors are trained and evaluated. They are not references: each is an individual factory-calibrated mid-cost electrochemical sensor, not a certified reference instrument (Appendix C). This three-tier architecture (Table 2) enables a direct quantitative comparison of what each investment level delivers.

All seven low-cost nodes were co-located at the laboratory for several days prior to field distribution, as a pre-deployment commissioning phase to verify operational integrity and coherent data acquisition across units under identical ambient conditions.

### 2.2. Data Collection, Quality Control, and Preprocessing

Data was collected from 16 April 2025 to 15 February 2026 (10 months, UTC), with low-cost and mid-cost sensors logged every 30 s and LAQN reference stations at 15 min averages. Table 3 reports the record counts and missing rates at each source. For the analysis, every source is averaged in 15-min intervals, and each 15-min value is called an observation.

All sources end between 11 and 15 February 2026. Each source stopped on a different date within this range: Nodes 3 and 5 were withdrawn for laboratory maintenance on 11 and 12 February, motivated by the accumulated hardware and software failures of the preceding months, and the reference series ends with the close of the study download window on 15 February. Gaps longer than 24 h occurred at the low-cost nodes and in the reference series. At Node 3 the largest were 12 d in November and 24 d between November and December; at Node 5, 24 d between October and November and 15 d in December. In the reference series, TH2 ${\mathrm{NO}}_{2}$ has a gap of 10.7 d between January and February 2026, and TH7 ${\mathrm{PM}}_{2.5}$ one of 15.7 d in January 2026. The low-cost gaps reflect hardware failures (including operating-system crashes), vandalism, and progressive low-cost sensor failures toward the end of the period.

**Figure 2:**
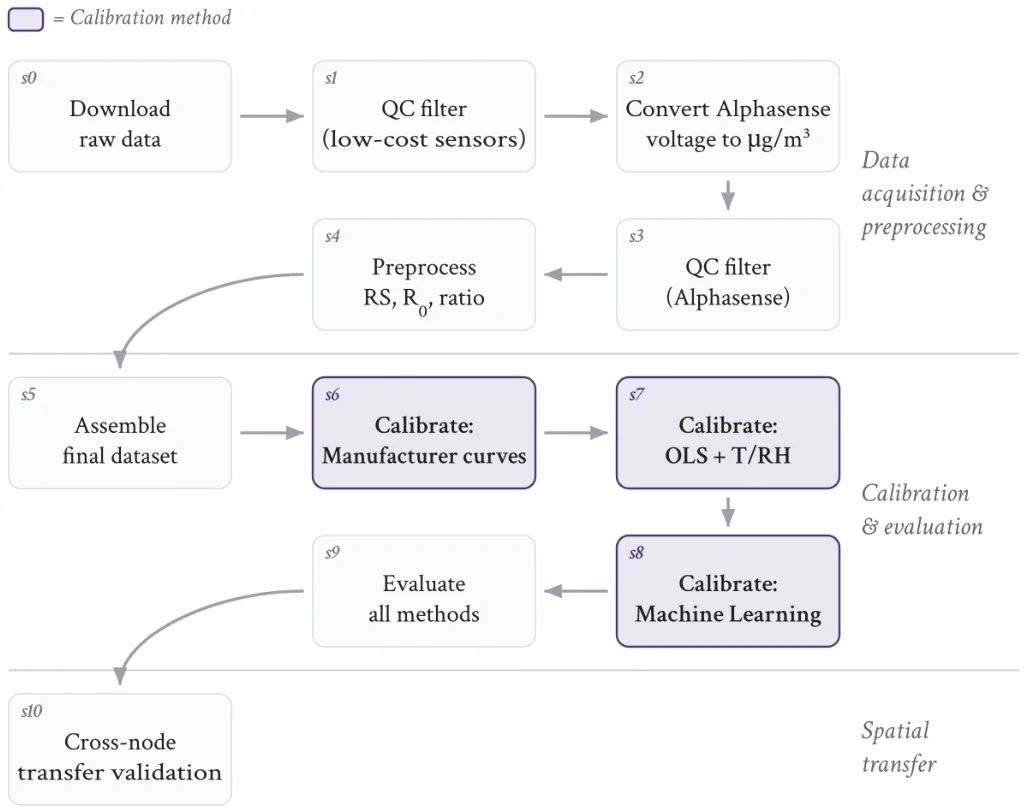
Data processing pipeline applied identically at both nodes: top row = data acquisition and preprocessing; middle row = dataset assembly, three calibration methods and evaluation; bottom = cross-node transfer.

An 11-stage pipeline (Figure 2) processes the raw streams identically at both nodes. The first phase acquires and preprocesses the data: raw records are downloaded; quality control is applied separately to the low-cost and Alphasense streams; the Alphasense raw voltages are converted to μg/m^3^ using AAN-803-05 Algorithm 1 [25], an Alphasense-provided algorithm that uses temperature to compensate for baseline drift via a WE − AE (working electrode minus auxiliary electrode) differential correction; and the metal oxide signals are transformed into the normalized ratio used for calibration (Section 2.3). The second phase assembles the merged dataset and applies three calibration methods of increasing complexity (manufacturer curves, Section 2.4; OLS regression with temperature and relative humidity covariates, Section 2.5; and Machine Learning, Section 2.6, which can incorporate the OLS baseline as an input feature), before evaluating all methods on identical metrics (Section 2.8). The third phase is the bidirectional cross-node transfer (Section 2.7).

Low-cost QC applies five filters in sequence and flags invalid values as NaN (Not a Number). Each filter acts only on the channels named in its label:

1. **Saturation (metal oxide channels):** analog-to-digital converter (ADC) raw values of 0 or 1023, and O_3_ voltages outside 0–4.95 V.
2. **Stuck readings (metal oxide channels):** ≥ 10 identical consecutive samples.
3. **Spikes (metal oxide channels):** Hampel filter with a half-window of h = 20 samples (a centered window of 41 samples) and a threshold of k = 4.0 median absolute deviations (MAD).
4. **SPS30 operational range (${\mathrm{PM}}_{2.5}$ and ${\mathrm{PM}}_{10}$):** values outside 0–1000 μg/m^3^ (the SPS30’s specified measurement range, not a universal physical bound on PM), followed by the Hampel filter of step 3.
5. **lEnvironmental range (temperature and relative humidity):** temperature outside −10 to 70 ^∘^C, or relative humidity outside 0–100%.

Alphasense QC applies three filters in sequence to each electrochemical channel:

1. **Physical bounds** per pollutant.
2. **Hampel filter** with the same parameters as the low-cost QC (h = 20, k = 4.0 MAD).
3. **Thermal-instability mask:** |ΔT| > 5 ^∘^C over 30 min triggers a 60-min recovery window during which data are blanked. These thresholds are implementation parameters of this pipeline, set because thermal transients can temporarily destabilize the sensor signal [25] and temperature affects the diffusion rate of electrochemical sensors even with auxiliary-electrode compensation [26].

### 2.3. Dynamic $R_{0}$: Addressing Baseline Drift in Low-Cost MOS

In low-cost MOS sensors, gas concentration is computed from the normalized resistance ratio in (1), where $R_{S}$ is the sensor resistance, $R_{0}$ its value in clean air, and the subscript corr denotes correction for temperature and relative humidity against the manufacturer table (referenced to 20 ^∘^C and 55% relative humidity). $R_{S}$ is itself recovered from a voltage divider (2), with the sensor in series with a fixed load resistor $R_{L}$ (=100 kΩ for CO, 47 kΩ for ${\mathrm{NO}}_{2}$, 1 kΩ for O_3_) and $V_{\mathrm{out}}$ read across $R_{L}$ by the ADC:

(1) $$\mathrm{Ratio}=R_{S,\mathrm{corr}}/R_{0}$$

(2) $$R_{S}=R_{L}\cdot \left(V_{\mathrm{cc}}-V_{\mathrm{out}}\right)/V_{\mathrm{out}}$$

$R_{0}$ is normally fixed before deployment by averaging $R_{S}$ over a few days in clean air. In the field, however, the true $R_{0}$ drifts continuously over weeks [22,27], a phenomenon known as *baseline drift*, and a fixed $R_{0}$ then biases every downstream calibration step.

**Figure 3:**
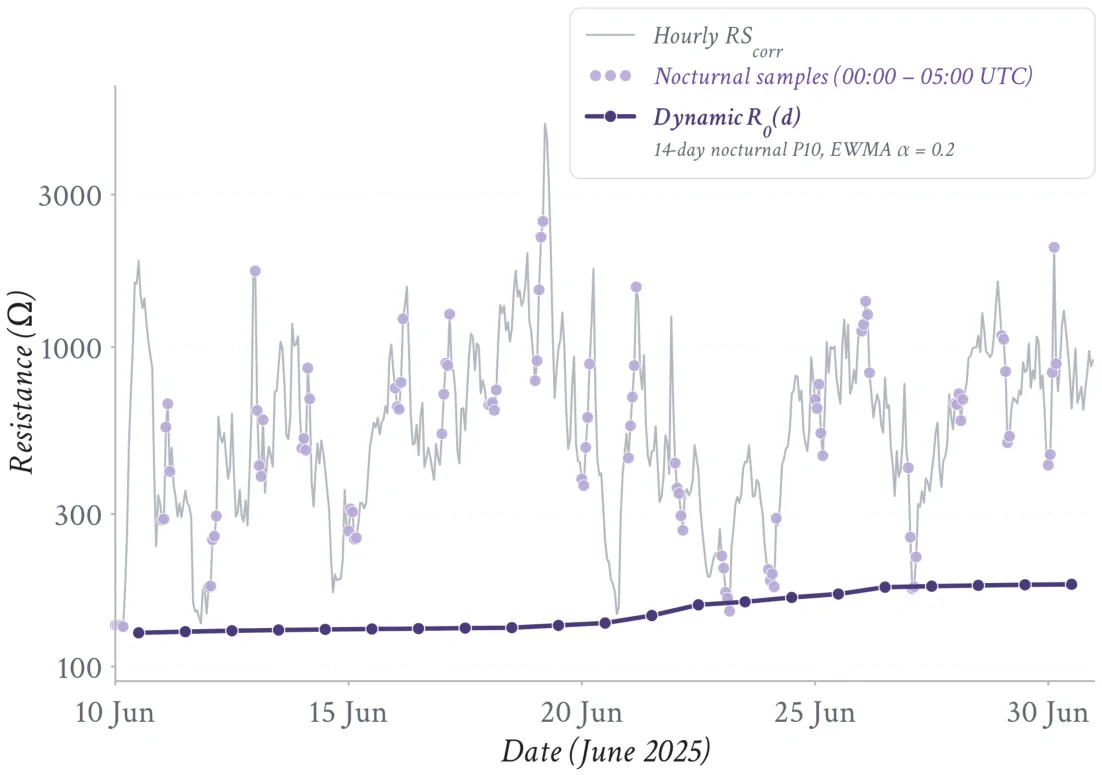
Dynamic $R_{0}$ for the GM-102B ${\mathrm{NO}}_{2}$ sensor at Node 3, 10–30 June 2025.

This study re-estimates $R_{0}$ each day from $R_{S,\mathrm{corr}}$ values measured at night, when traffic is at its minimum and the sensor signal reaches its lowest values. These lowest nightly values are taken here as the practical proxy for clean air. The EWMA smoothing and 14-day percentile window described below absorb atypical nights, such as winter temperature inversions that trap pollutants overnight and briefly break the proxy. Figure 3 shows $R_{0}$(d) following the lower edge of the nightly samples and rising slowly over June 2025. The procedure is:

**(1) Nocturnal subset.** $R_{S,\mathrm{corr}}$ is aggregated to 15-min means over 00:00–04:59 UTC, the window of minimum traffic emissions at urban roadside sites and therefore the closest empirical proxy to clean air.

**(2) Rolling percentile.** For each day *d* over a trailing 14-day window W(d) (a growing window of min(d,14) days is used during the first 13 days):

(3) $$\begin{array}{c} R_{0,\mathrm{raw}}\left(d\right)=P_{90}\left(R_{S,\mathrm{corr}}|W\left(d\right)\right)\\ \mathrm{for}\;\mathrm{reducing}\;\mathrm{gases}\;\left(\mathrm{CO}\right) \end{array}$$

(4) $$\begin{array}{c} R_{0,\mathrm{raw}}\left(d\right)=P_{10}\left(R_{S,\mathrm{corr}}|W\left(d\right)\right)\\ \mathrm{for}\;\mathrm{oxidizing}\;\mathrm{gases}\;({\mathrm{NO}}_{2},\;O_{3}) \end{array}$$

The asymmetry follows from n-type MOS physics: reducing gases lower $R_{S}$, so clean air sits in the upper tail; oxidizing gases raise $R_{S}$, so clean air sits in the lower tail. $P_{90}$/$P_{10}$ balances proximity to the true baseline (more extreme percentiles are noisier) against residual-contamination bias (less extreme percentiles underestimate).

**(3) EWMA smoothing.**

(5) $$\begin{array}{c} R_{0}\left(d\right)=\alpha \cdot R_{0,\mathrm{raw}}\left(d\right)+\left(1-\alpha \right)\cdot R_{0}\left(d-1\right)\\ \alpha =0.2 \end{array}$$

The 0.8 weight on the prior estimate gives an effective averaging horizon of ∼5 days, making $R_{0}\left(d\right)$ robust to single-night perturbations while still tracking the slow drift. The recursion is initialized by setting $R_{0}$($d_{1}$) = $R_{0,\mathrm{raw}}$($d_{1}$) on the first day with a valid estimate.

**(4) Insufficient-data handling.** If W(d) contains fewer than 20 valid 15-min nocturnal periods (=5 h), the last valid $R_{0}$ is carried forward for up to 30 consecutive days; beyond that, $R_{0}$ = NaN and the affected measurements are excluded. This occurs during initial warm-up, extended outages (Section 2.2), or aggressive QC flagging.

The method requires no reference instrument, only co-located temperature and relative humidity measurements, and could be applied post-hoc to other MOS sensor time series that have a nocturnal traffic minimum.

### 2.4. Calibration Method 1: Manufacturer Curves

The manufacturer calibration curves, extracted from sensor datasheets [18,28,29], convert the$R_{S}$/$R_{0}$ ratio to gas concentration using log-log (CO, O_3_) or linear (NO_2_) interpolation. These curves were designed for laboratory conditions far above urban ambient levels: 1–1000 ppm for the GM-702B (CO), 0–10 ppm for the GM-102B (NO_2_), and 0.01–1 ppm (10–1000 ppb) for the MQ131 (O_3_). Ambient concentrations measured at the study sites by the co-located references and factory-calibrated mid-cost electrochemical sensors over the 10-month observation period were approximately 0.1–1 ppm for CO (median ≈0.1 ppm, peaks to ≈1 ppm; Alphasense CO-B4), 2–30 ppb for NO_2_ (LAQN reference), and 5–70 ppb for O_3_ (median ≈20 ppb; Alphasense OX-B431). The manufacturer calibration thus operates at the extreme lower end of its designed range for CO and NO_2_, and within the lowest decile of the range for O_3_, serving in this study as the baseline representing what a sensor delivers with no field calibration whatsoever.

### 2.5. Calibration Method 2: OLS Regression

OLS regression with environmental covariates is widely adopted as a field calibration method for low-cost sensors [12,13,26], and the evaluation framework of the European Committee for Standardization Technical Specification CEN/TS 17660-1:2021 [30] uses regression-based metrics to assess sensor performance against reference instruments. OLS is interpretable, reproducible, and provides the linear calibration against which ML improvements can be quantified. Hayward et al. [12] argued that for electrochemical and optical sensors, simple linear models perform comparably to complex non-parametric methods. The comparison of ML against OLS therefore tests whether this conclusion extends to metal oxide sensors.

The OLS model has the form:

(6) $$C=\beta_{0}+\beta_{1}\cdot \mathrm{signal}+\beta_{2}\cdot T+\beta_{3}\cdot RH$$

where the signal is the ratio for gaseous pollutants or the raw SPS30 ${\mathrm{PM}}_{2.5}$ reading, and *T* and *RH* are the co-located SHT45 temperature and relative humidity measurements. Each regression is fitted only on the training set of Section 2.6, hence the name train-only OLS used below, and its coefficients are applied unchanged to the test set. Table 4 lists the OLS regressions for each pollutant.

### 2.6. Calibration Method 3: Machine Learning

Five models represent three families of algorithms (bagging, gradient boosting and neural networks), a family being a group of models that share the same learning principle. The literature finds these three families consistently competitive for tabular regression in low-cost sensor calibration. Random Forest (RF) represents bagging and is the most widely used model for this task [11,19,26,31]. Extreme gradient boosting (XGBoost) and the light gradient boosting machine (LightGBM) represent gradient boosting [32]. Neural networks are represented by a deep neural network (DNN), a multi-layer perceptron with two hidden layers, and by a long short-term memory (LSTM) network that reads 24-h input windows, a model with which Bachechi et al. [33] outperformed manufacturer baselines over two years of NO, ${\mathrm{NO}}_{2}$ and O_3_ monitoring.

Three feature configurations are evaluated for each model and target, a target being one sensor to be calibrated together with the reference or mid-cost sensor whose concentration its model predicts (Table 4):

- **Config A (ML pure):** The model receives only measurements of the node, without the OLS prediction that Configs B and C add. For every low-cost gas target, the 12 features (Table A1, Appendix A) are the raw signals of the three metal oxide sensors (GM-102B, GM-702B, and MQ131), temperature, relative humidity, the absolute humidity derived from them, the raw VOC signal, the SPS30 ${\mathrm{PM}}_{2.5}$ readings, and four cyclic time features (sine/cosine of hour and day of week). For the ${\mathrm{PM}}_{2.5}$ target, Config A has 10 features instead of 12: the same list without the three metal oxide signals and with the SPS30 ${\mathrm{PM}}_{10}$ readings added. The gas signals are raw ADC counts or voltages, not the $R_{S}$/$R_{0}$ ratio of Section 2.3. Appendix A also documents how the features are prepared.
- **Config B (OLS+ML stacking):** The model receives the Config A features plus the OLS prediction as an additional feature. This stacking approach provides the ML algorithm with a physically informed prior. This gives 13 features for gases and 11 for ${\mathrm{PM}}_{2.5}$.
- **Config C (OLS correction):** The model receives only the OLS prediction, temperature, relative humidity, absolute humidity, and temporal features (8 features in total). This configuration tests whether ML can improve upon OLS using only the OLS output and environmental context.

The experimental design has five targets per node: the four low-cost sensors (${\mathrm{PM}}_{2.5}$, CO, NO_2_ and O_3_), which are the object of this study, and the mid-cost Alphasense ${\mathrm{NO}}_{2}$, the only mid-cost sensor with a co-located reference instrument. Each target is modeled with the three configurations and the five models, that is, 15 combinations per target and 75 per node. The hyperparameter search (Table A2) and the model selection described below are run separately for each target, the mid-cost ${\mathrm{NO}}_{2}$ included, so every model is selected among the 15 combinations of its own target. At Node 3, the six LSTM combinations for ${\mathrm{NO}}_{2}$ (two ${\mathrm{NO}}_{2}$ targets × three configurations) produce no prediction, because the TH2 ${\mathrm{NO}}_{2}$ reference never provides 24 h of consecutive values (Appendix B). Node 3 therefore has 69 results, and Node 5 has 75 (Section 3.2).

Data are split by time, not at random. Of the 29,376 observations per node, those up to 31 October 2025 00:00 UTC form the training set (19,009, 64.7%) and those from 1 November 2025 00:00 UTC form the test set (10,272, 35.0%). The 95 observations of 31 October 2025 after 00:00 UTC belong to neither set. Observations with a missing feature or reference value are dropped per target, so the effective counts differ by target (Table A3).

Under this split, the OLS input of Configs B and C is the prediction of the train-only OLS of Section 2.5, so no reference value from the test period enters any ML input. The same OLS is the linear calibration reported in Section 3, where the manufacturer curves, the OLS and the ML models are scored on the same test observations of each target (Table A3). Of those observations, an LSTM predicts only the ones preceded by a complete 24 h window (Appendix B).

Hyperparameters are optimized with RandomizedSearchCV on the training data only. The search scores each sampled configuration by the negative root mean square error (RMSE) over the five folds of a TimeSeriesSplit. It samples 100 configurations for RF, LightGBM and XGBoost, 40 for the DNN and 20 for the LSTM, with a random seed of 42. Random search finds equally good or better models than grid search at a fraction of the compute cost in the high-dimensional hyperparameter spaces used here (5–9 parameters per model) [34]. The search spaces, network architectures, early-stopping rules, seeds, and software versions are listed in Appendix B.

**Figure 4:**
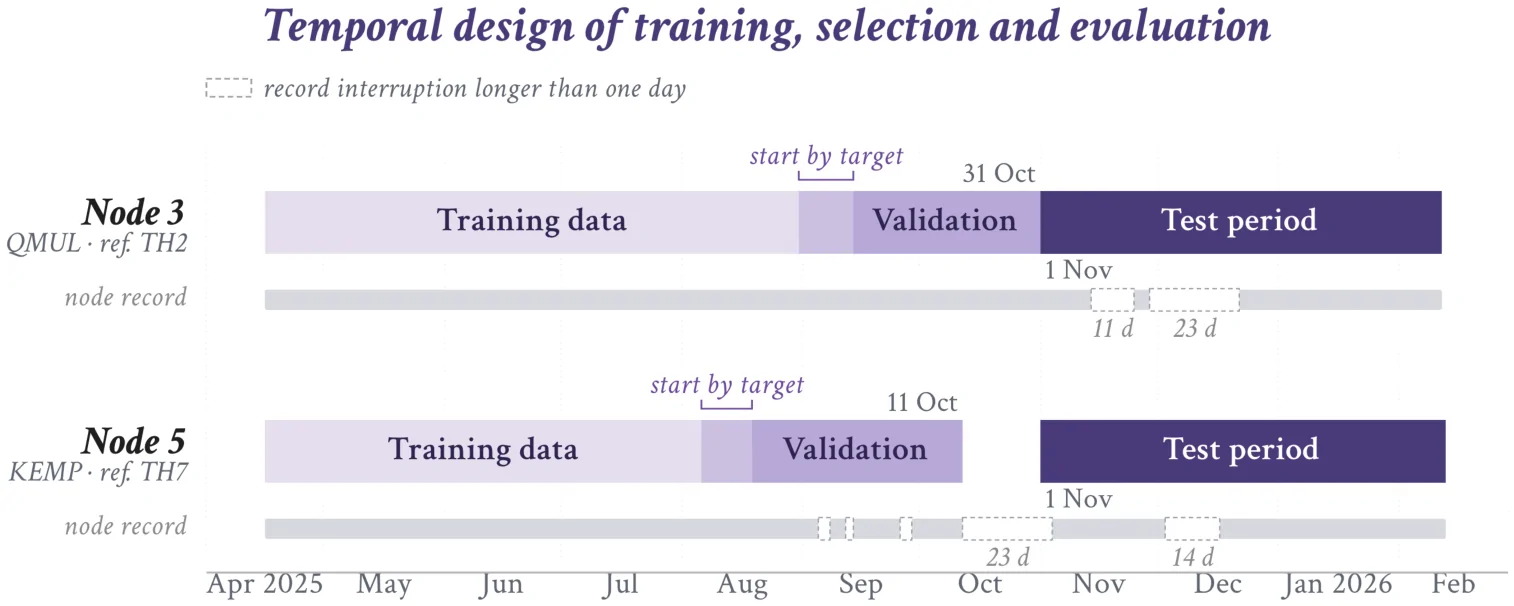
Training data, validation block and test period at both nodes, with the interruptions of the node record.

Model selection does not use the test period. For each target and node, the 15 combinations (5 models × 3 configurations) are compared on a validation block formed by the last 25% of the training observations of each combination. Figure 4 shows the three blocks at both nodes and the interruptions of the node record longer than one day. Each combination is refitted with its selected hyperparameters on the earlier training observations, with the OLS input of Configs B and C refitted on the same observations, and is scored on the block. The combination with the lowest validation RMSE is the model selected for that target (Table A4), called the validation-selected model below (the ’locked’ model of a pre-specified selection). Its test performance, which played no role in the choice, is the result reported for each target in Section 3.2 (Table 5, column ‘Selected Model’). The test $R^{2}$ of all combinations is still shown in Section 3.2 to describe the search space, and the maximum over the 15 combinations is reported as an upper bound labeled as such. For the cost-performance comparison of Section 4.1, the same validation block also decides between the train-only OLS (Section 2.5) and the validation-selected ML model of each sensor. The method with the lower validation RMSE is the one reported for that tier (Table A5).

The combinations of a target are not scored on the same observations (Appendix B, Effective observations per target). The model selection and the test comparison between the selected model and the train-only OLS are therefore repeated on the observations that all combinations of a target predict.

**Ablation of the inputs.** To measure what the raw sensor signal contributes to the ML calibration, Random Forest models are also trained on five reduced input sets. Set 1 contains temperature, humidity, and the cyclic time features alone, and set 2 adds the SGP40 VOC signal and the SPS30 ${\mathrm{PM}}_{2.5}$. Set 3 contains the raw channel of the target sensor alone, and set 4 the three metal oxide channels alone. Set 5 is Config A without the target sensor, which isolates what that sensor adds to all the other inputs. Config A is the reference for this comparison because it contains all of these inputs and no OLS prediction. Each reduced model uses the search protocol above and is scored on the Config A observations of its target, so its test $R^{2}$ is comparable with that of Config A (Section 3.3, Table 6). ${\mathrm{PM}}_{2.5}$ is included as a comparison case for a sensor with a near-linear response. The features of each set are listed in Appendix B, and each feature is described in Table A1 (Appendix A).

### 2.7. Cross-Node Transfer Protocol

The ultimate objective of calibrating low-cost sensors is to deploy them where reference stations do not exist, thereby expanding spatial coverage. If a model trained at one reference site can be applied to identical sensors at sites without reference, then a single reference station could serve as a calibration hub for an entire neighborhood network.

To evaluate this, the study exploits the fact that Nodes 3 and 5 are each co-located with an independent government reference station. This design allows measurement of the degradation that would occur when transferring a model to a site without a reference. In a real deployment, the transferred model would be applied to Nodes 1, 2, 4, 6, and 7, which lack any co-located reference.

**Figure 5:**
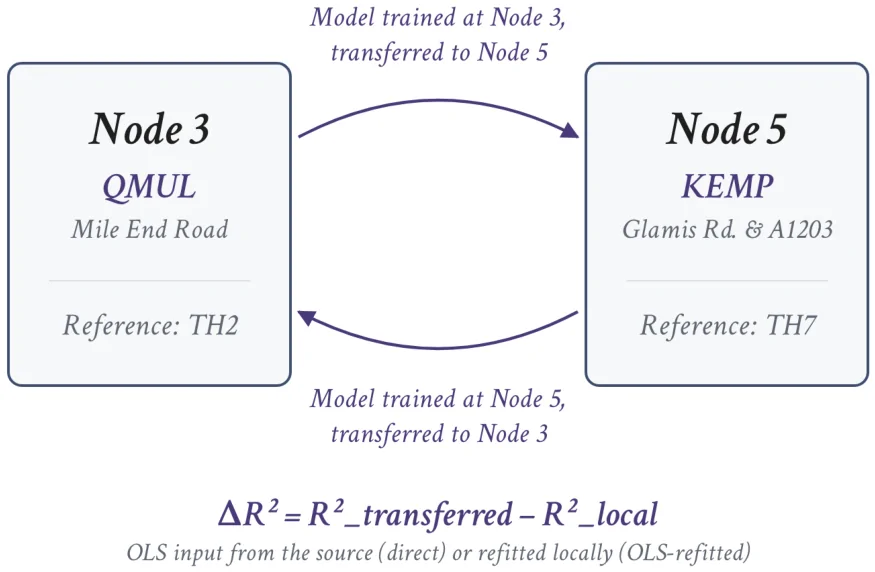
Bidirectional cross-node transfer protocol.

The transfer protocol is bidirectional (Figure 5): models trained on Node 3 data (QMUL, reference TH2) are evaluated on Node 5 data against TH7, and vice versa. The node where a model is trained is called the source node, and the node to which it is transferred is called the destination node. The destination reference is used only to score the transferred predictions. Transfer degradation is quantified as $\Delta R^{2}$ (transferred minus local, computed before rounding). Three scenarios are distinguished by how much information from the destination node they use:

- **lDirect transfer.** The validation-selected model of the source node (Section 2.6) is applied at the destination as trained at the source, without any change. For Configs B and C the OLS input is computed with the OLS coefficients fitted at the source node, so no destination reference value enters the inputs. This is the only scenario available at a site without a co-located reference, and it is the main result of Section 3.5.
- **OLS-refitted transfer.** The same model, but the OLS input of Configs B and C is refitted with the destination reference on the destination training observations (Section 2.6), while the ML model keeps the weights learned at the source. This requires a co-location at the destination, at least temporarily. The scenario measures what a local refit of the linear input recovers without retraining the model.
- **Destination-selected transfer.** The model is selected with the reference data of the destination node, information that a real deployment would not have. For each gas and direction, three source candidates are transferred with the OLS-refitted input: the validation-selected model, the model with the highest source test $R^{2}$, and the simplest model within 0.05 $R^{2}$ of that maximum, in the order RF, then XGBoost and LightGBM, then DNN, then LSTM. The candidate with the highest destination $R^{2}$ is reported. This scenario shows which model families can transfer when the best candidate is known in advance; it is not a procedure that a deployment can apply.

### 2.8. Evaluation Metrics

All calibration methods are evaluated using the coefficient of determination ($R^{2}$), normalized RMSE (nRMSE, normalized by the reference mean), RMSE, mean absolute error (MAE), mean bias error (MBE), and Pearson correlation coefficient (r). $R^{2}$ and r quantify how well the sensor tracks reference variability ($R^{2}$ additionally penalizing offset and slope); RMSE and MAE measure absolute error magnitude (RMSE weights large errors quadratically, MAE is more robust to outliers); MBE, the mean of predicted minus reference values, captures systematic over-prediction (positive) or under-prediction (negative); and nRMSE rescales RMSE by the reference mean to enable cross-pollutant comparison. These metrics are reported without external classification thresholds, consistent with recent practice in the low-cost sensor literature [10,11,12,19,20]. The European CEN/TS 17660-1:2021 [30] classifies sensors into three classes based on relative expanded uncertainty (REU), but this requires certified reference instrument uncertainty data not available for the LAQN stations used in this study.

## 3. Results

### 3.1. Performance by Cost Tier and Calibration Method

**Figure 6:**
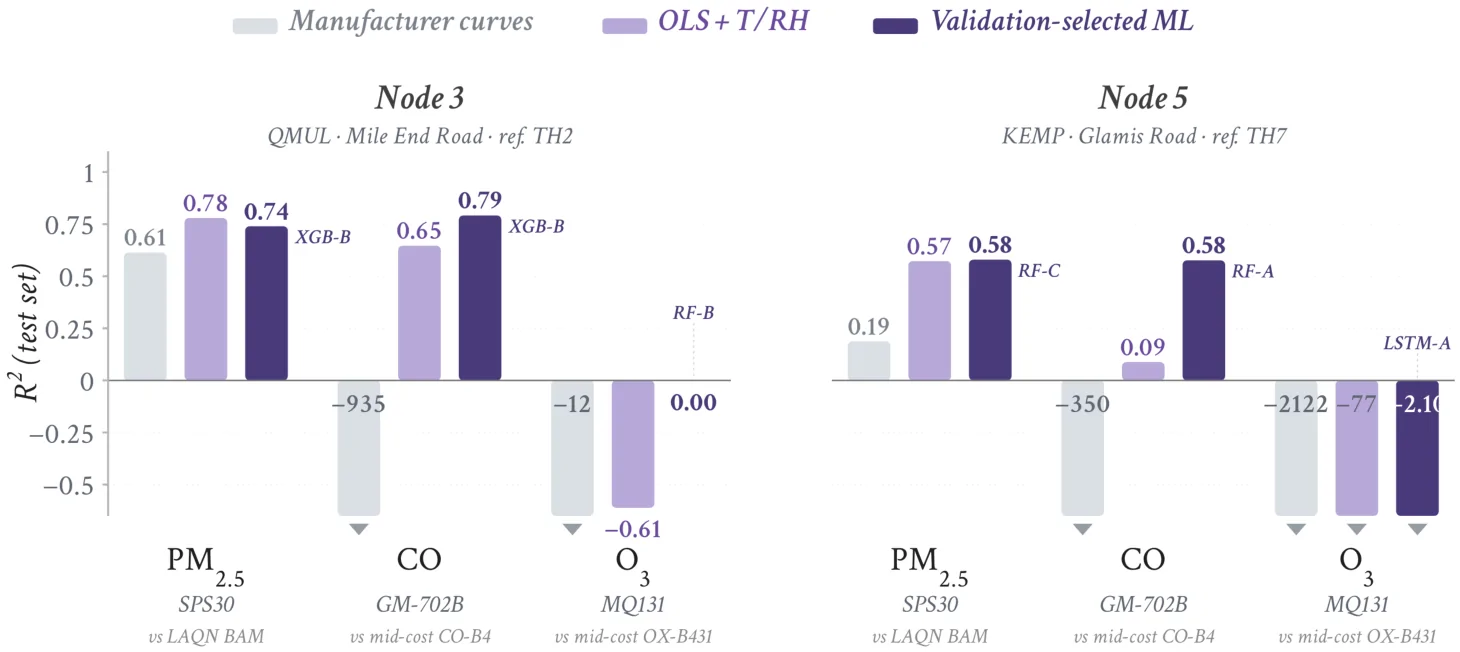
Test $R^{2}$ per calibration method for three low-cost sensors at Nodes 3 and 5. Truncated bars (▾) are labeled with their value. XGB: XGBoost; T/RH: temperature and relative humidity.

**Figure 7:**
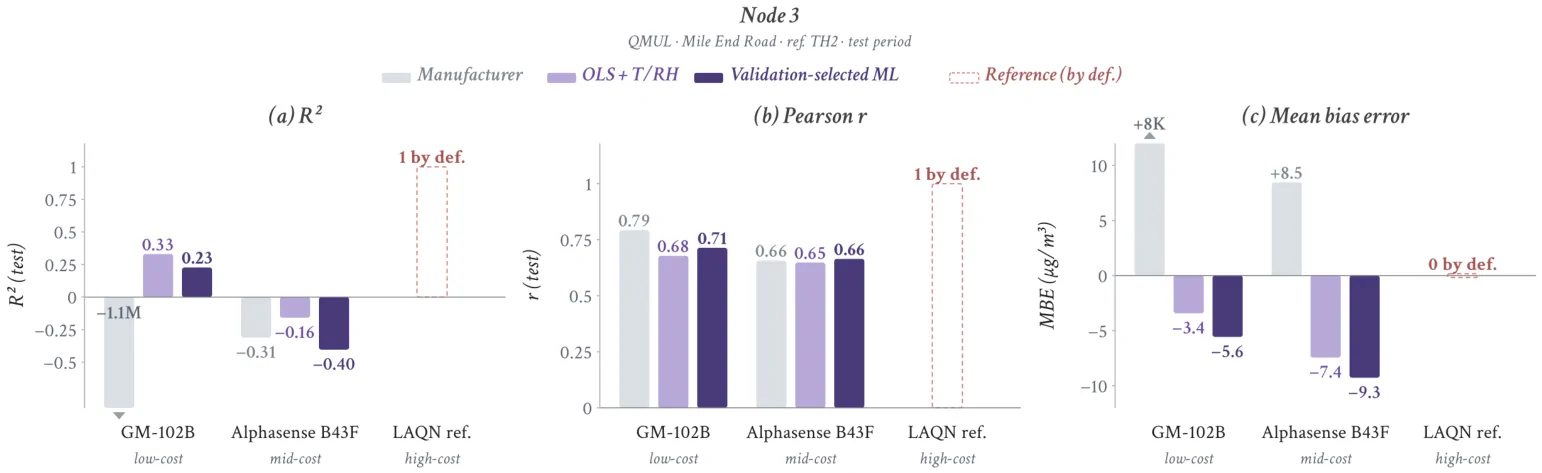
Test $R^{2}$, Pearson r and mean bias error (MBE) per cost tier for ${\mathrm{NO}}_{2}$ at Node 3. Bars marked with an arrow are truncated and labeled with their value (M = 10^6^, K = 10^3^). T/RH: temperature and relative humidity.

Figure 6 reports $R^{2}$ on the test data for the three low-cost sensors common to both nodes (SPS30, GM-702B, MQ131), with references as listed in Table 4. The three calibration methods are the manufacturer curves (Section 2.4), the train-only OLS with temperature and relative humidity covariates (Section 2.5) and the validation-selected ML model of Table 5 (Section 2.6). ${\mathrm{NO}}_{2}$ is reported separately in Figure 7 because it is the only pollutant for which low-, mid-, and high-cost instruments are co-located. ${\mathrm{PM}}_{2.5}$ has no mid-cost tier because electrochemical sensing, which defines that tier, applies to gases only, and a mid-cost PM instrument would rely on the same optical scattering principle as the low-cost SPS30. Its informative contrast is therefore the low-cost optical sensor against the beta-attenuation reference.

**${\mathrm{PM}}_{2.5}$ (SPS30).** Manufacturer calibration is site-dependent: Node 3 retains a usable signal ($R^{2}=0.61$) while Node 5 does not (0.19, Figure 6). The train-only OLS raises the $R^{2}$ to 0.78 at Node 3 and 0.57 at Node 5, and the validation-selected ML model does not improve on it at either node (Table 5).

**CO (GM-702B).** Manufacturer curves give an $R^{2}$ of −935 at Node 3 and −350 at Node 5 (Figure 6), because the datasheet covers 1–1000 ppm while ambient CO sits at 0.1–1 ppm (Section 2.4). OLS recovers a usable signal at Node 3 but almost none at Node 5. The ML model selected on the validation block improves on it at both nodes, to $R^{2}=0.79$ and 0.58 (Table 5), in agreement with the co-located factory-calibrated mid-cost electrochemical sensor (Appendix C). These are, at the time of writing, the first published field calibration results for the GM-702B.

**O_3_ (MQ131).** Manufacturer and OLS calibration both fail at both nodes, and every point-based ML model (RF, XGBoost, LightGBM, DNN) also yields a negative $R^{2}$ (Figure 6). A point-based model predicts each observation from the inputs of that observation only, whereas the LSTM reads the preceding 24 h. The validation-selected models give $R^{2}=0.00$ at Node 3 (RF-B) and −2.10 at Node 5 (LSTM-A). Only the LSTM reaches a positive test $R^{2}$ at Node 3 (Config A, 0.14), and only as the upper bound of Table 5. On the observations that the LSTM predicts, the selected RF-B reaches 0.17 (Section 3.2). The hypothesis that the LSTM’s 24-h window leverages temporal autocorrelation rather than the MQ131 signal itself is examined in Section 4.2.

Once calibrated in the field, the low-cost GM-102B gives a higher test $R^{2}$ than the mid-cost Alphasense ${\mathrm{NO}}_{2}$-B43F at both Nodes 3 and 5 (Figure 7 shows Node 3; Table 5 gives both). At Node 3 the $R^{2}$ of the low-cost sensor is positive with the train-only OLS and with the validation-selected ML model, whereas that of the mid-cost sensor is negative with its factory calibration, with the OLS and with ML (Figure 7a). The two sensors correlate with the reference almost equally, with Pearson r values that differ by less than 0.07 (Figure 7b). What differs is the bias, whose cause is examined in Section 4.3: under each of its calibrations, the mean bias error of the mid-cost sensor has a larger absolute value than that of the calibrated low-cost sensor (Figure 7c).

**${\mathrm{NO}}_{2}$(GM-102B).** Manufacturer $R^{2}$ collapses by more than five orders of magnitude (Figure 7). OLS recovers a weak but positive signal at both nodes, and the ML model selected on the validation block improves on it at Node 5 but falls below it at Node 3 (Figure 7, Table 5). The highest test $R^{2}$ among the 15 combinations at Node 3 (0.58, DNN-A) is not reached by the selected model: this is the clearest case in the study of a result that selection on the test would have inflated. As with the GM-702B, no prior field calibration of the GM-102B has been published.

Across the three MOS gases, manufacturer curves are unusable in field conditions: beyond the off-range issue analyzed in Section 2.4, datasheet curves describe an averaged rather than per-unit response, so per-unit field calibration is required. The same pattern was independently reported by Hong et al. [35] for the Sensirion SGP30 MOS sensor, whose manufacturer calibration showed a mean normalized bias of +228% to +473% against reference VOC concentrations over 10–12 months of field deployment.

**Figure 8:**
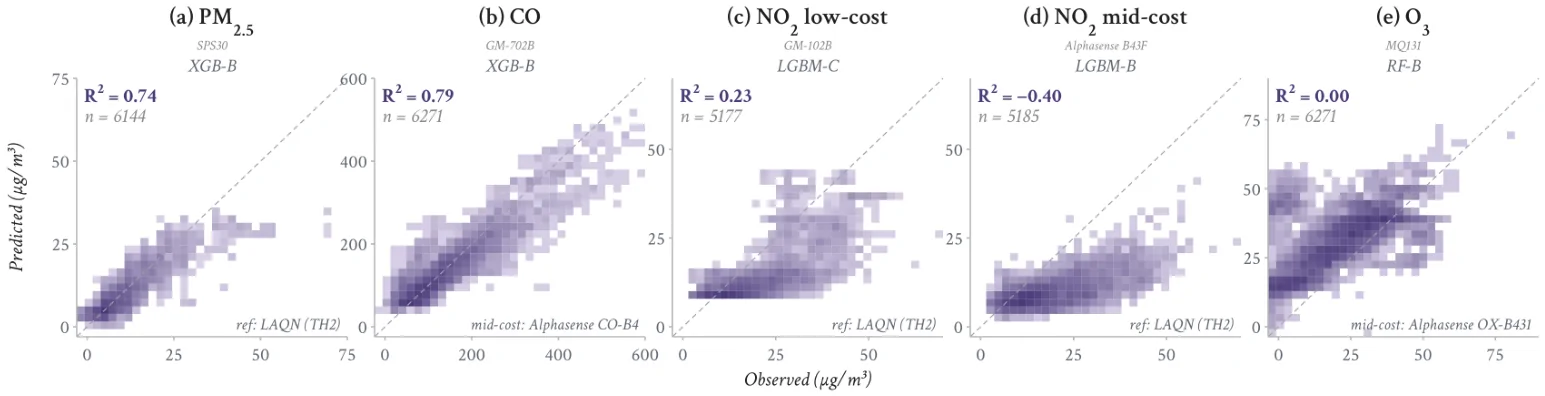
Density of observed vs predicted concentrations at Node 3 for the validation-selected ML model per pollutant (Table 5); reference (${\mathrm{PM}}_{2.5}$ and ${\mathrm{NO}}_{2}$) and mid-cost sensor (CO and O_3_) labeled in the panels. Dashed line: 1:1. LGBM: LightGBM; XGB: XGBoost.

Figure 8 visualizes the resulting fit at Node 3: tight density along the 1:1 line for ${\mathrm{PM}}_{2.5}$ and CO, broader dispersion for ${\mathrm{NO}}_{2}$, and visible temporal structure in the O_3_ residuals.

### 3.2. ML Model Comparison

**Figure 9:**
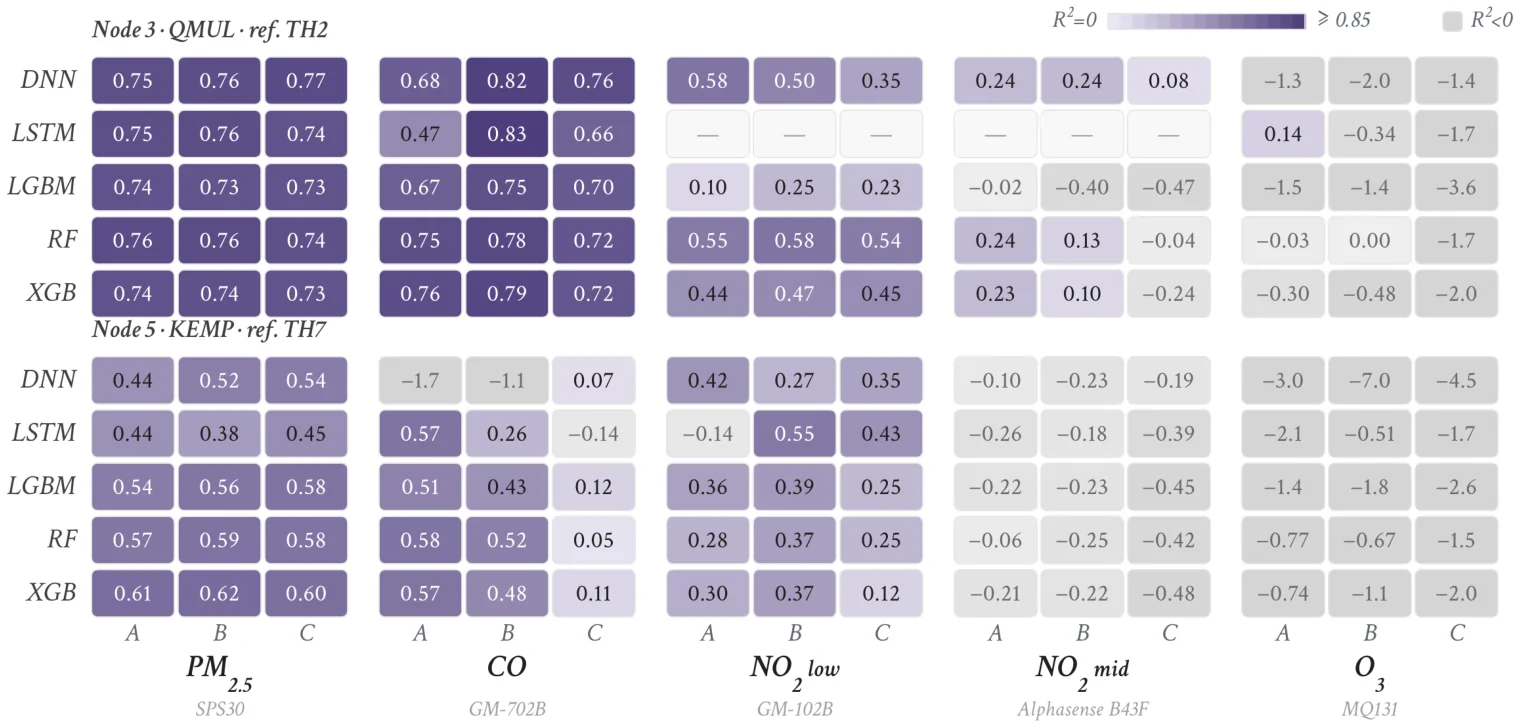
Test $R^{2}$ of the 75 ML combinations per node (5 models × 3 feature configurations × 5 targets). Gray cells: negative $R^{2}$. Dashes: LSTM combinations that cannot be scored (Section 2.6). LGBM: LightGBM; XGB: XGBoost.

For the 75 ML combinations per node (Figure 9), Random Forest (RF) is the most consistently robust architecture: highest mean test $R^{2}$ across the four low-cost pollutants at both nodes (0.37 N3, 0.07 N5), with a median train-to-test $R^{2}$ ratio of 1.3 (N3) and 1.7 (N5). The validation-selected model per target and node is listed in Table 5 next to the highest test $R^{2}$ among the 15 combinations of its target (5 models × 3 feature configurations, Figure 9). The two coincide for CO at Node 5 and differ by less than 0.05 $R^{2}$ for ${\mathrm{PM}}_{2.5}$ at both nodes and for CO at Node 3. For ${\mathrm{NO}}_{2}$ and O_3_ the selected model falls well below the maximum. The reason is that the best candidates reach almost the same validation RMSE, so the selection rule cannot tell them apart. At Node 3 the selected LightGBM-C and the second-ranked RF-C differ by 0.5% in validation RMSE, yet their test $R^{2}$ differ by 0.3. A parsimony rule that selects the simplest model within 2% of the lowest validation RMSE (Appendix B) would choose a different model in three cases at Node 3, including this one (Table A4). For the mid-cost Alphasense ${\mathrm{NO}}_{2}$-B43F, the model selected from the same grid gives a negative test $R^{2}$ at both nodes (Table 5).

**On the common observations, the selected model changes for two of the ten targets and the comparison with the train-only OLS for none.** The selection gives the same model as Table 5 for eight targets (Table A4, column Common observations). At Node 5, the ${\mathrm{NO}}_{2}$ model changes from XGBoost-B (test $R^{2}$ 0.373) to LightGBM-A (0.360) and the mid-cost ${\mathrm{NO}}_{2}$ model from LSTM-C (−0.392) to XGBoost-B (−0.222). On the common test observations, the selected model exceeds the OLS in the same seven targets as on its own observations. For O_3_ at Node 3, the selected RF-B reaches 0.17 on the observations that the LSTM predicts, above the 0.14 of the LSTM-A.

**No single family dominates the validation-selected models, and the performance of the DNN is site-specific.** Gradient boosting (XGBoost, LightGBM) is selected for four of the eight low-cost targets and RF for three. The DNN, which reaches the highest test $R^{2}$ at Node 3 for ${\mathrm{PM}}_{2.5}$ and ${\mathrm{NO}}_{2}$ (Table 5), is never selected because its validation RMSE is not the lowest. The LSTM is selected only for O_3_ and the mid-cost ${\mathrm{NO}}_{2}$ at Node 5, where every model fails. The same Config B that yields $R^{2}=0.82$ for CO at Node 3 collapses to $R^{2}=-1.09$ at Node 5 for the DNN; tree-based architectures show no comparable cross-site swing.

**Overfitting varies by family.** Median train-to-test $R^{2}$ ratios stay below 2.1 for RF and XGBoost at both nodes (maximum 2.02, XGBoost at Node 5), but LightGBM reaches 7.3 for ${\mathrm{NO}}_{2}$ at Node 3, and the LSTM on O_3_ at Node 3 reaches 6.9 (train $R^{2}=0.95$, test $R^{2}=0.14$); the latter is examined in Section 4.2.

**Table 5:** ML calibration model selected on the validation block (Section 2.6) per pollutant and node, with its test $R^{2}$, its validation RMSE (μg/m^3^) and the highest test $R^{2}$ among the 15 combinations of the same target. Notation: MODEL-CONFIG, where CONFIG is the feature configuration of Section 2.6; LGBM: LightGBM; XGB: XGBoost.

Pollutant	Node	Selected Model ($R^{2}$)	Val. RMSE	Highest $R^{2}$ Among 15
${\mathrm{PM}}_{2.5}$	3	XGB-B (0.740)	2.82	DNN-C (0.769)
${\mathrm{PM}}_{2.5}$	5	RF-C (0.580)	2.44	XGB-B (0.619)
CO	3	XGB-B (0.792)	49.0	LSTM-B (0.834)
CO	5	RF-A (0.577)	57.2	RF-A (0.577)
${\mathrm{NO}}_{2}$	3	LGBM-C (0.228)	8.67	DNN-A (0.579)
${\mathrm{NO}}_{2}$	5	XGB-B (0.373)	7.34	LSTM-B (0.548)
${\mathrm{NO}}_{2}$ (mid-cost)	3	LGBM-B (−0.404)	9.88	DNN-B (0.242)
${\mathrm{NO}}_{2}$ (mid-cost)	5	LSTM-C (−0.392)	6.38	RF-A (−0.062)
O_3_	3	RF-B (−0.003)	17.4	LSTM-A (0.139)
O_3_	5	LSTM-A (−2.103)	17.3	LSTM-B (−0.509)

### 3.3. Feature Configurations: A vs B vs C

The selection on the validation block (Section 2.6) kept the OLS prediction among the model inputs only where the OLS itself performs well. Where the train-only OLS reaches a test $R^{2}$ of 0.33 or more, the selected configuration includes the OLS prediction (Config B or C). This happened for ${\mathrm{PM}}_{2.5}$ at both nodes and for CO and ${\mathrm{NO}}_{2}$ at Node 3. Where the OLS gives a test $R^{2}$ near zero or below, the selected configuration uses the raw sensor signals alone (Config A). This happened for CO and O_3_ at Node 5 (Table 5, Figure 6 and Figure 7).

This pattern has two exceptions, ${\mathrm{NO}}_{2}$ at Node 5 and O_3_ at Node 3. In both, the selected configuration includes the OLS prediction even though the OLS test $R^{2}$ is poor, 0.18 for ${\mathrm{NO}}_{2}$ and negative for O_3_. The cause is a near tie in validation: the best configuration without the OLS prediction (Config A) had a validation RMSE only 0.4% higher for ${\mathrm{NO}}_{2}$ and 3.4% higher for O_3_.

**Ablation of the inputs.** Without the metal oxide signals, the ML calibration of CO and ${\mathrm{NO}}_{2}$ loses 0.21 to 0.40 $R^{2}$ with respect to Config A at both nodes (Table 6, Section 2.6). With temperature, humidity, and the cyclic time features alone (set 1), the Random Forest gives an $R^{2}$ of 0.06 or below for every target. Adding the VOC and ${\mathrm{PM}}_{2.5}$ readings (set 2) raises CO to 0.52 at Node 3 and 0.28 at Node 5, so part of the CO agreement comes from pollutants that rise and fall together with CO, not from the GM-702B itself. Removing only the target sensor from Config A (set 5) lowers the test $R^{2}$ by 0.05 to 0.07 for CO at both nodes and for ${\mathrm{NO}}_{2}$ at Node 3, and by 0.22 for ${\mathrm{NO}}_{2}$ at Node 5.

The target signal alone and the three metal oxide signals alone (sets 3 and 4) are not sufficient for the metal oxide gases: they stay below Config A for CO at both nodes and for ${\mathrm{NO}}_{2}$ at Node 3, and give a negative $R^{2}$ for CO at Node 5. The exception is ${\mathrm{NO}}_{2}$ at Node 5, where the three MOS channels alone reach the $R^{2}$ of Config A (0.30 against 0.28). For ${\mathrm{PM}}_{2.5}$ the environmental features add at most 0.11 to the $R^{2}$ of the SPS30 signal alone.

**Table 6:** Test $R^{2}$ of Random Forest models trained on reduced input sets, on the Config A observations of each target and node. The input sets are defined in Appendix B. For ${\mathrm{PM}}_{2.5}$, Config A without the target signal equals the set of the fourth column. T/RH: temperature and relative humidity.

Target	Node	T/RH/Time	+ VOC, ${\mathrm{PM}}_{2.5}$	Target Signal	All Sensor Signals	Without Target Signal	Config A
CO	3	0.04	0.52	0.34	0.60	0.68	0.75
CO	5	0.06	0.28	−0.13	−0.48	0.52	0.58
${\mathrm{NO}}_{2}$	3	−0.17	0.34	0.23	0.22	0.49	0.55
${\mathrm{NO}}_{2}$	5	−0.47	−0.12	0.27	0.30	0.06	0.28
${\mathrm{PM}}_{2.5}$	3	−0.34	−0.25	0.77	0.76	−0.25	0.76
${\mathrm{PM}}_{2.5}$	5	−0.21	−0.18	0.46	0.46	−0.18	0.57

### 3.4. Monthly Diagnosis over 10 Months

**Figure 10:**
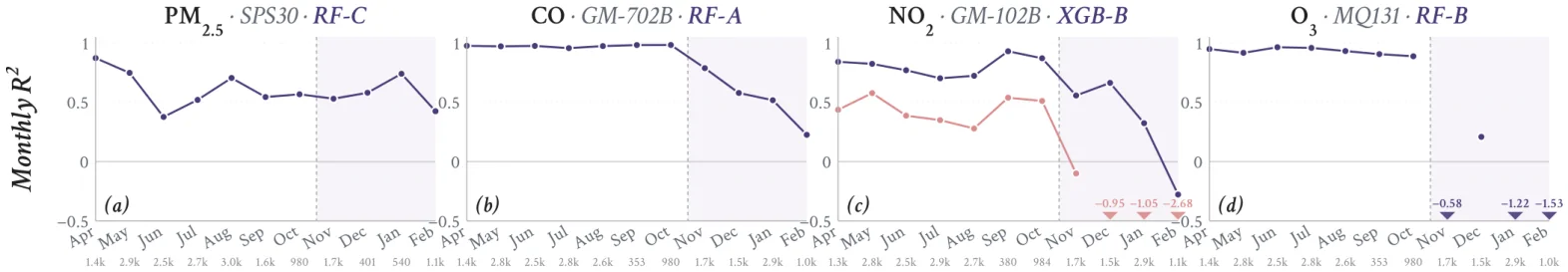
Monthly $R^{2}$ at Node 5, April 2025 to February 2026, of the validation-selected model of each low-cost sensor (indigo): (**a**) ${\mathrm{PM}}_{2.5}$, SPS30, RF-C; (**b**) CO, GM-702B, RF-A; (**c**) ${\mathrm{NO}}_{2}$, GM-102B, XGB-B, and the mid-cost ${\mathrm{NO}}_{2}$-B43F calibrated with the train-only OLS (salmon); (**d**) O_3_, MQ131, RF-B, the model selected at Node 3 (Section 3.4). Shading: test period (November to February); unshaded: training period (April to October), whose last 25% of observations form the validation block (Figure 4); numbers below the axis: valid observations per month; ▾: values below the axis; XGB: XGBoost.

To diagnose how the calibration performance changes over the deployment period, the validation-selected model of each low-cost sensor (Table 5) was applied month by month to the full 10-month record without retraining (Figure 10, Node 5). Two properties of this diagnosis constrain its reading. First, the months from April to October are the training period, so their $R^{2}$ describes the fit to data the model has seen, which tree-based models fit closely. Only November to February test the model on unseen data. Second, because of the outages of Section 2.2, the number of valid 15-min observations differs by month, from 353 (CO, September, Node 5) to 2960 (CO, January, Node 3). The monthly $R^{2}$ values therefore rest on different sample sizes, shown under each panel of Figure 10. Whether a decline within the test months also reflects aging of the sensor is discussed in Section 4.5. For O_3_ the RF-B model selected at Node 3 was used at both nodes, because the LSTM selected at Node 5 predicts only the observations preceded by 24 h without gaps, which leaves at most 88% of a month and none in September 2025. At Node 5 the test $R^{2}$ of RF-B is 0.16 below the LSTM upper bound of Table 5.

**The SPS30 (${\mathrm{PM}}_{2.5}$) is the most stable sensor.** In the test months, its monthly $R^{2}$ stays between 0.42 and 0.74 at Node 5 (Figure 10a) and between 0.65 and 0.78 at Node 3, so the calibration keeps describing the test data.

**The GM-702B (CO) declines at Node 5 but not at Node 3.** At Node 5 the $R^{2}$ is 0.96 to 0.98 in the training months and falls through the test months, from 0.79 in November to 0.23 in February (Figure 10b). At the more sheltered Node 3 site there is no sustained decline: 0.74 to 0.91 in the training months, 0.54 in November (the month with the 12 d outage), and 0.76 to 0.93 from December to February. The CO decline is therefore a one-site observation.

**The GM-102B (${\mathrm{NO}}_{2}$) is the only sensor whose calibration declines at both sites.** At Node 5 the $R^{2}$ falls from 0.56 in November to −0.28 in February (Figure 10c). The September value (0.93) rests on 380 valid 15-min observations, about one eighth of a complete month, because the metal oxide channels of Node 5 returned few valid readings that month. It is not a reliable peak. At Node 3 the $R^{2}$ falls from 0.54 in November to −1.06 in February.

**The train-only OLS is as stable as the selected ML model over the test months.** In every test month at both nodes, the OLS and the ML model differ by less than 0.2 $R^{2}$ for ${\mathrm{PM}}_{2.5}$, and the ML model stays above the OLS for CO. For ${\mathrm{NO}}_{2}$ the OLS and the ML model decline together at Node 3, whereas at Node 5 the ML model is above the OLS by 0.32, 0.44, and 0.02 from November to January. In February both are negative for ${\mathrm{NO}}_{2}$ at both nodes.

**The MQ131 (O_3_) calibration was not reliable at either node.** At Node 5 the RF-B model fits its training months at an $R^{2}$ of 0.89 or more and is negative in every test month except December (Figure 10d). At Node 3 the training $R^{2}$ is above 0.94, and the test months alternate in sign (−0.86, 0.78, 0.52, and −0.38 from November to February). The two positive months do not make the calibration reliable, because over the whole test period the $R^{2}$ is 0.00 (Table 5). A high training fit with a test fit that fails or changes sign suggests that the model learned temporal and meteorological correlates of O_3_ rather than a response of the MQ131 to O_3_ that agrees with the mid-cost sensor (Section 4.2).

**The mid-cost Alphasense ${\mathrm{NO}}_{2}$ calibration also fails in the test months.** Calibrated with the train-only OLS, the sensor does not exceed a monthly $R^{2}$ of 0.6 in any training month at either node (Figure 10c). In the test months its $R^{2}$ is negative from December at Node 3 and from November at Node 5. The mid-cost sensor therefore loses its calibration on the same horizon as the low-cost GM-102B.

### 3.5. Cross-Node Transferability

**Figure 11:**
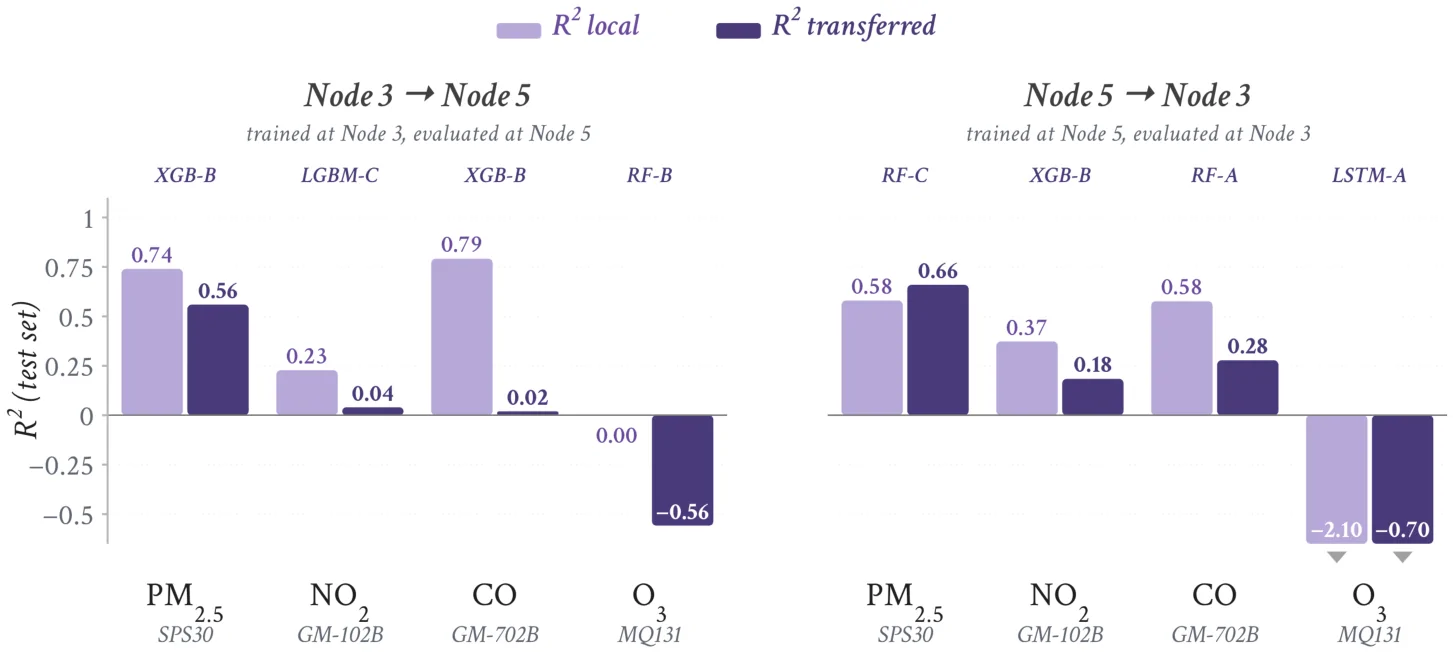
Test $R^{2}$ of the validation-selected model at the source node (pastel) and at the destination node (indigo) in the direct transfer scenario (Table 7). Bars below $R^{2}=-0.65$ are truncated (▾). LGBM: LightGBM; XGB: XGBoost.

Transfer of the validation-selected models of Table 5 between the two nodes, in both directions and in the three scenarios of Section 2.7, shows where spatial coverage extension is feasible (Table 7, Figure 11).

**${\mathrm{PM}}_{2.5}$ transfers in both directions.** In direct transfer, ${\mathrm{PM}}_{2.5}$ loses less than 0.2 $R^{2}$ in one direction and gains slightly in the other (Table 7). At either destination the transferred model performs close to the model selected locally at that node (Table 5). Refitting the OLS input at the destination recovers only part of the small loss in one direction and nothing in the other.

**The linear calibration transfers as well as the ML model.** Applied at the destination with the coefficients fitted at the source, the train-only OLS reaches $R^{2}=0.57$ and 0.74 for ${\mathrm{PM}}_{2.5}$ in the two directions of Table 7. These values are within 0.04 of the OLS fitted at the destination itself (Section 3.1) and are equal to or above the transferred ML model (Table 7). For ${\mathrm{NO}}_{2}$ the transferred OLS keeps $R^{2}=0.21$ and 0.25, above the transferred ML model and within 0.08 of the destination OLS. For CO, the transferred OLS is negative, and for O_3_ both methods fail.

**${\mathrm{NO}}_{2}$ does not transfer with source information only.** In direct transfer, the ${\mathrm{NO}}_{2}$ models keep at most a fifth of the variance at the destination ($R^{2}$≤0.18, Table 7), well below the models selected locally at each node (Table 5). Refitting the OLS input at the destination changes $R^{2}$ by at most 0.04 (Table 7), and even the destination-selected transfer does not reach the local values.

**CO and O_3_ fail in both directions.** The transferred CO models fall to $R^{2}$≤0.28 at the destination, and refitting the OLS input there recovers only part of the loss (Table 7). O_3_ has no usable local signal to begin with (Section 3.1), and the transferred O_3_ models are negative in both directions (Table 7).

**The destination-selected scenario raises $R^{2}$ by less than 0.1 in five of the eight cases of Table 7.** In this scenario the destination reference chooses the transferred model (Section 2.7), and the gain is measured against the OLS-refitted scenario, or direct transfer, where Table 7 gives n/a. The three exceptions are ${\mathrm{NO}}_{2}$ from Node 3 to Node 5 and O_3_ in both directions (Section 4.2). Even with this choice, ${\mathrm{NO}}_{2}$, CO and O_3_ stay at $R^{2}$≤0.28 at the destination: the failure of the gas models is not an artifact of model selection. For ${\mathrm{NO}}_{2}$ and CO the destination-selected values also stay below the models calibrated locally (Table 5), so a local calibration after a temporary co-location gains more than any choice among source models.

In these data, the ML models transferred to a site without a reference only where the following three conditions held:

1. **Local $R^{2}$ above approximately 0.5.** A model that has not learned a strong signal cannot transfer one: the two ${\mathrm{PM}}_{2.5}$ models, both above 0.5 at the source, transferred, and the two ${\mathrm{NO}}_{2}$ models, both below 0.4, did not.
2. **Identical sensor model and type at the destination**, so the relation between sensor signal and concentration learned at the source applies.
3. **A standardized, government-grade reference at training**, not an individual sensor. Government-grade analyzers are calibrated to national standards and therefore interchangeable across sites; individual Alphasense electrochemical units are not. ${\mathrm{PM}}_{2.5}$, trained against government references, transferred. CO and O_3_, trained against individual Alphasense units, did not. O_3_ also fails the first condition, and for CO other explanations remain possible (Section 4.4). ${\mathrm{NO}}_{2}$ was also trained against government references but did not transfer because its source $R^{2}$ was below 0.5, the first condition above.

## 4. Discussion

### 4.1. The Cost-Performance Trade-Off

**Figure 12:**
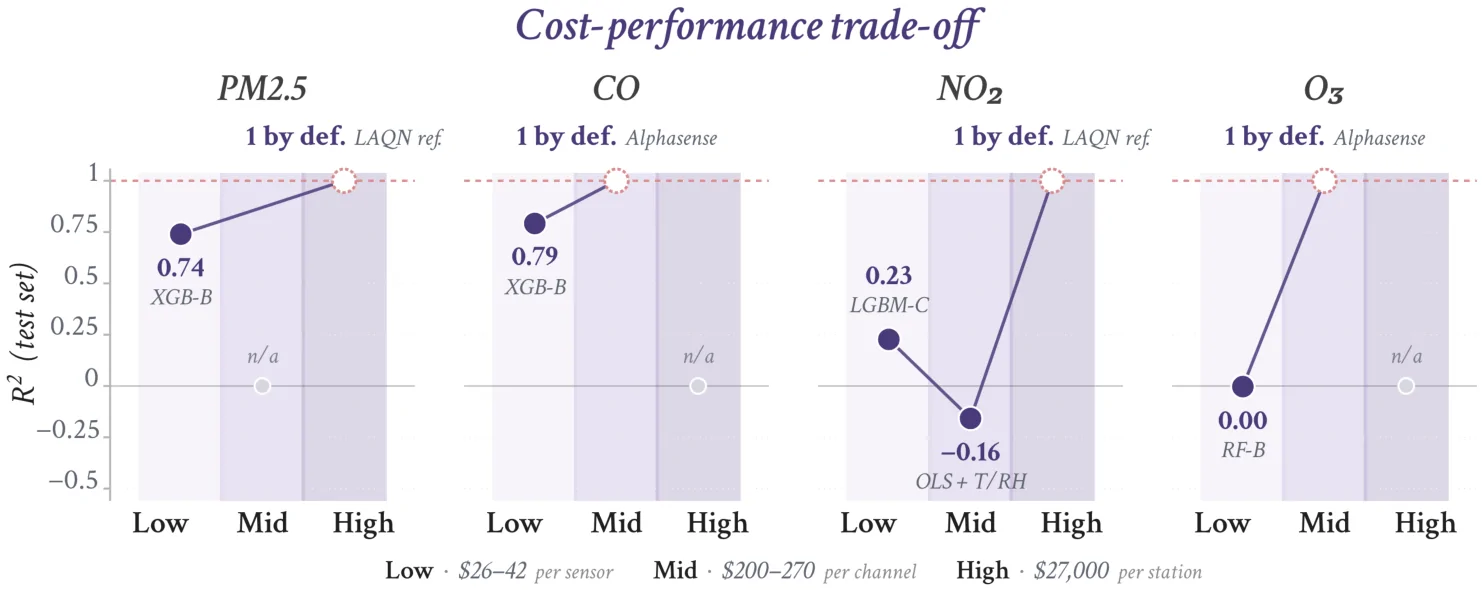
Test $R^{2}$ per pollutant and cost tier at Node 3 for the method selected on the validation block (Section 2.6), labeled at each point (LGBM: LightGBM; XGB: XGBoost). Reference tier and mid-cost tier for CO and O_3_, at $R^{2}=1$ by definition; n/a = no co-located instrument; T/RH: temperature and relative humidity.

Whether low-cost sensors can deliver useful air quality data depends on the pollutant (Figure 12). The results of Section 3.1 group into four distinct regimes.

**${\mathrm{PM}}_{2.5}$: smallest performance gap.** The SPS30 reaches $R^{2}=0.78$ with the train-only OLS and 0.74 with the validation-selected ML model at Node 3 (Section 3.1) and is the only low-cost sensor whose calibration keeps describing the test data at both nodes (Section 3.4). At roughly 1/700 the cost of the reference station (Table 2), this is the closest a low-cost sensor in this study comes to its high-cost benchmark. Optical scattering relates more directly to particle mass than MOS surface chemistry, which is consistent with this smaller gap.

**CO: agreement with the mid-cost sensor.** The GM-702B + ML agrees with the co-located Alphasense CO-B4 at $R^{2}=0.79$ (Node 3, Section 3.1) despite costing approximately 5–7 times less per sensor. In the Random Forest of Table 6, the model without the GM-702B keeps most of the $R^{2}$ of Config A, whereas the GM-702B alone reaches 0.34 at Node 3 and a negative $R^{2}$ at Node 5 (Section 3.3). The agreement is therefore obtained with the node inputs together, not with the GM-702B alone. No public CO reference exists at either site (Section 4.5), which limits the cost saving in two ways. First, each deployment must install its own factory-calibrated mid-cost electrochemical sensor to train the model. Second, the trained model does not transfer to a sensor at another site (Section 3.5 and Section 4.4). The cost saving therefore depends on the availability of public reference infrastructure for the target pollutant, not on the sensor price alone.

**${\mathrm{NO}}_{2}$: a six-fold cost increase does not improve the signal.** Under each of its three calibrations, the mid-cost Alphasense ${\mathrm{NO}}_{2}$-B43F gives a lower test $R^{2}$ than the field-calibrated GM-102B at both nodes (Section 3.1, Table 5), for the reason examined in Section 4.3. A countervailing benefit of the mid-cost tier is service life: Alphasense specifies an operating life of more than 24 months (months until 50% of the original signal, 24-month warranted) for the ${\mathrm{NO}}_{2}$-B43F [36]. In this study, however, the calibration of the deployed unit stopped describing the test data in the same months as the low-cost ${\mathrm{NO}}_{2}$ calibration (Section 3.4), so this study does not test the longer specified service life.

**O_3_: low-cost data were not usable in this study.** The MQ131’s response covers 10–1000 ppb, but ambient O_3_ stays in the bottom 10% of that range, which leaves insufficient signal for point-based calibration (Section 4.2). The LSTM’s $R^{2}=0.14$ at Node 3, the upper bound of Table 5, most likely captures the site’s daily O_3_ pattern rather than the sensor’s signal itself, and no O_3_ model transfers (Section 4.4). At the concentrations observed here, reliable ambient O_3_ monitoring requires a different sensor technology (electrochemical or UV absorption), and the cost-performance question must be answered at a higher tier.

### 4.2. Why O_3_ Partially Fails and What the LSTM Reveals

Two physical reasons can explain why the MQ131 fails at ambient concentrations. First, ambient O_3_ stays in the lowest 10% of the calibration range (Section 4.1), where the manufacturer curve spans $R_{S}$/$R_{0}$ ratios of only 1.2 to 2.8 [18], a small signal relative to the noise. Second, the MQ131 also responds to other oxidizing gases, such as ${\mathrm{NO}}_{2}$ [18], and its baseline drifts (Section 2.3). Badura et al. [15] already reported the largest inter-unit variability for the MQ131 (Section 1.2), and here no point-based ML model (RF, XGBoost, LightGBM, DNN) recovers a signal at ambient concentrations (Section 3.1).

The LSTM is the only model that reaches a positive test $R^{2}$ at Node 3 (0.14, the upper bound of Table 5; the selected RF-B gives 0.00). In the observations that the LSTM predicts, however, the selected RF-B reaches 0.17 (Section 3.2). Two observations support the hypothesis that the LSTM learns the daily O_3_ pattern rather than the sensor’s signal. First, its training $R^{2}$ of 0.95 falls to 0.14 on the test months (Section 3.2), and the tree-based O_3_ models of the monthly diagnosis are negative in five of the eight test months of the two nodes (Section 3.4). Second, what the LSTM learns transfers between the sites: the Node 3 model gives $R^{2}=-0.01$ at Node 5, above every model trained at Node 5, and the Node 5 LSTM-B gives 0.17 at Node 3, above every model trained at Node 3 (destination-selected transfer, Table 7). A calibration of a specific sensor unit transfers worse than it works locally (Section 3.5); a model of a daily pattern shared by two sites 1.6 km apart does not. The 24-h sliding window therefore most likely captures the temporal regularity of ambient O_3_, not a sensor-to-concentration mapping.

A further source of uncertainty is the factory-calibrated mid-cost electrochemical sensor used for O_3_: the OX-B431 responds to O_3_ and ${\mathrm{NO}}_{2}$ together, and its ${\mathrm{NO}}_{2}$ response is subtracted using the co-located ${\mathrm{NO}}_{2}$-B43F (Appendix C), whose bias (Section 3.1) propagates to the O_3_ values of the OX-B431.

### 4.3. When Is ML Justified over a Linear Calibration?

For the optical SPS30, the linear calibration is sufficient, which confirms the finding of Hayward et al. [12] that linear models perform as well as non-linear ones for electrochemical and optical sensors. On the same test data (Section 3.1), the train-only OLS with temperature and relative humidity covariates equals or exceeds the validation-selected ML model at both nodes. Over the test months, its monthly $R^{2}$ stays within 0.2 of that of the ML model (Section 3.4), and it transfers between the sites (Section 3.5). It needs no model selection and no training beyond a linear fit, so the extra complexity of ML is not justified for ${\mathrm{PM}}_{2.5}$. For the mid-cost Alphasense ${\mathrm{NO}}_{2}$-B43F the comparison is not informative: the linear calibration and the validation-selected ML model both give negative test $R^{2}$ at both nodes (Table 5, Figure 7a), so neither can be said to suffice.

For the metal oxide sensors, whether ML is justified over the linear calibration depends on the gas. For CO (GM-702B) ML is justified at both sites: it gains +0.15 and +0.49 $R^{2}$ over the OLS at Nodes 3 and 5 (Section 3.1), stays above it in every month (Section 3.4), and at Node 5 is the only method that recovers a signal. For ${\mathrm{NO}}_{2}$ (GM-102B), the ML model falls below the OLS at Node 3 and exceeds it at Node 5, where its margin narrows to 0.02 by January (Section 3.4). At Node 3, nine of the twelve ${\mathrm{NO}}_{2}$ combinations with a result exceed the OLS on the test period, but the validation block selects none of them (Section 3.2). The linear calibration is therefore the default for this sensor: it gives a positive $R^{2}$ at both sites without any model selection, whereas the sign of the ML gain depends on the site.

The lower performance of the mid-cost Alphasense ${\mathrm{NO}}_{2}$-B43F (Section 4.1) is not caused by the calibration method. What differs from the low-cost sensor is its bias, of 7 to 9 μg/m^3^ in absolute value, while the two correlations with the reference are almost equal (Section 3.1, Figure 7). The test months are colder than any training month (Section 4.5). The baseline of the electrochemical cell depends on temperature and is corrected with factors tabulated by the manufacturer (Appendix C), whereas the metal oxide baseline is re-estimated daily (Section 2.3). Whether an adaptive correction of that baseline would recover the mid-cost performance is left for future work.

The OLS prediction used as an ML input is not what makes the ML calibration work. The selection on the validation block keeps this input where the OLS itself performs well (Section 3.3). However, the gain of ML over the environmental inputs comes from the metal oxide signals, not from the OLS input. Config A, which has no OLS input, already exceeds the inputs without these signals (set 2) by 0.21 to 0.40 $R^{2}$ (Table 6). The pattern of Section 3.3 is therefore a tendency observed in these data, not a rule for choosing the inputs.

### 4.4. Transferability: Expanding Spatial Coverage Without Reference Stations

Transfer is meant to calibrate low-cost nodes where no reference station exists, with a model trained where one does (Section 2.7). The outcome differs by pollutant.

${\mathrm{PM}}_{2.5}$ is the strongest case: $|\Delta R^{2}|$≤0.18 in both directions in direct transfer (Section 3.5). The SPS30 response is close to linear (Section 4.3), which is consistent with a calibration that holds at another site. ${\mathrm{NO}}_{2}$ does not transfer in the same scenario (Table 7), which suggests that the GM-102B calibration depends on conditions of the training site that the model does not separate from the signal. A ${\mathrm{NO}}_{2}$ calibration with this sensor therefore still requires a co-location at each site.

CO and O_3_ fail, probably for different reasons. For CO, the proposed explanation is training against an individual mid-cost electrochemical sensor with its own factory calibration (Table A6), whereas a standardized reference such as LAQN is interchangeable across sites. Differences between the two GM-702B units and site-specific relations between CO and the other inputs (Section 3.3) are alternatives that one unit per node cannot exclude (Section 4.5). The O_3_ failure is different: the MQ131 gives no usable signal even locally (Section 3.1), and Section 4.2 discusses possible physical causes. Without a public reference for CO or O_3_ in this network, pure model transfer remains an open challenge.

The contrast with prior work is informative. Winter et al. [20] obtained $R^{2}=0.88$ for O_3_ by transferring electrochemical sensor calibrations at 17–29 km in BEA${\mathrm{CO}}_{2}$N. The metal oxide ${\mathrm{NO}}_{2}$ sensor here retains at most $R^{2}=0.18$ at 1.6 km with the transferred ML models (0.25 with the linear calibration), and the optical SPS30 0.56 to 0.66. Electrochemical sensors transferred substantially better in that study, at approximately 5–7 times the per-sensor cost (Section 4.1). In the present study, however, the mid-cost ${\mathrm{NO}}_{2}$ sensor did not perform better locally (Section 4.1), so portability of calibration cannot be attributed to the price tier alone.

### 4.5. Limitations

Two caveats concern the instruments against which the low-cost sensors are evaluated. First, no reference instrument for CO or O_3_ exists at either site (Section 2.1), so those results quantify the agreement between the low-cost sensor and a single co-located factory-calibrated mid-cost electrochemical sensor. Its uncertainty is known only from the factory calibration and the datasheet specifications (Appendix C), and the results cannot be compared directly with those for ${\mathrm{PM}}_{2.5}$ and ${\mathrm{NO}}_{2}$. Second, ${\mathrm{NO}}_{2}$ is the only pollutant for which all three cost tiers are co-located (Section 3.1), so the comparison between the low-cost and the mid-cost tier rests on one gas and one pair of sensors per site.

Three caveats concern the dataset. First, both sites are urban roadsides in East London, so transferability to other cities or climates is untested. Second, the dataset covers 10 months and a single winter (16 April 2025 to 15 February 2026). Every test month is colder and later than every training month, so the decline of Section 3.4 in the test months can reflect the change of season or the aging of the sensors, and this dataset cannot separate the two. Third, one unit of each sensor model was deployed per node, so inter-unit variability could not be quantified.

Three caveats concern the methods. First, only direct transfer shows what a site without a reference would obtain (Section 3.5), since the OLS-refitted scenario needs a temporary co-location at that site and the destination-selected scenario is only a diagnostic (Section 2.7). Second, the LSTM is the least explored model family, because its search sampled the fewest configurations (Table A2) and it gave no result for ${\mathrm{NO}}_{2}$ at Node 3 (Section 2.6). Third, the validation block that selects among the combinations (Section 2.6) was also part of the training data on which their hyperparameters were searched. This overlap can change which combination is selected, but not the reported test $R^{2}$, because the test data entered neither the search nor the selection.

## 5. Conclusions and Future Work

This study presents, at the time of writing, the first published field calibration of the GM-102B (${\mathrm{NO}}_{2}$) and GM-702B (CO) metal oxide sensors, and a systematic comparison of three sensor cost tiers under three calibration methods (manufacturer curves, OLS, ML) at two London sites over 10 months (Section 2.1, Table 2).

**What performance do low-cost sensors achieve?** With the validation-selected ML models (Section 2.6), three of the four low-cost sensors achieve a positive test $R^{2}$ for their target pollutant. Against the LAQN reference instruments, the SPS30 (${\mathrm{PM}}_{2.5}$) reaches $R^{2}=0.74$ and 0.58 at Nodes 3 and 5, and the GM-102B (${\mathrm{NO}}_{2}$) reaches 0.23 and 0.37. Against the co-located factory-calibrated mid-cost electrochemical sensor, the GM-702B (CO), with the other node signals, reaches 0.79 and 0.58 (Section 4.1). The MQ131 (O_3_) is the only sensor that fails point-based calibration entirely (Section 3.1 and Section 4.1). The only positive test $R^{2}$ for O_3_ is 0.14, from an LSTM at Node 3 that the validation block did not select (Table 5). It most likely reflects temporal regularity rather than the sensor’s chemoresistive signal (Section 4.2).

**How much does each calibration step improve performance?** Manufacturer curves of the metal oxide sensors are unusable at ambient concentrations (Section 3.1). On the same test data, the train-only OLS with temperature and relative humidity equals or exceeds the validation-selected ML model for ${\mathrm{PM}}_{2.5}$ at both sites and for ${\mathrm{NO}}_{2}$ at one site. ML improves on it for CO at both sites (+0.15 and +0.49 $R^{2}$) and for ${\mathrm{NO}}_{2}$ at the other site (Section 4.3). The mid-cost electrochemical ${\mathrm{NO}}_{2}$-B43F gives test $R^{2}$ values between −0.91 and 0.05 at the two nodes under its factory calibration, the train-only OLS, and the validation-selected ML model. The highest value among its 15 ML combinations, 0.24 at Node 3 (Table 5), was not selected on the validation block.

**Can mid-cost electrochemical sensors calibrate low-cost sensors where no reference exists?** Conditionally. The Alphasense CO-B4 enables the CO calibration ($R^{2}=0.79$), the only feasible path when no government CO reference exists in the network (Section 4.1). For ${\mathrm{NO}}_{2}$, the GM-102B with ML against the LAQN station outperforms the Alphasense ${\mathrm{NO}}_{2}$-B43F under any method (Section 4.1 and Section 4.3), so a standardized reference is preferable when available. For O_3_, the MQ131 fails irrespective of which reference is used (Section 4.2).

**How does calibration performance change over 10 months?** Among the low-cost sensors, the ${\mathrm{PM}}_{2.5}$ calibration keeps describing the test data at both nodes (test-month $R^{2}$ of 0.42 to 0.78). The ${\mathrm{NO}}_{2}$ calibration stops describing the test data at both nodes, and the CO calibration at one node, from November onwards, after about six months of deployment. O_3_ never produces a usable signal (Section 3.4). Whether the gas-sensor decline reflects the change of season between the training and the test months (recoverable by retraining) or hardware aging (requiring replacement) cannot be settled with this dataset. A record that includes the following spring would separate the two: if performance recovers when the season returns, the decline was seasonal, and if it does not, aging is the more likely cause.

**Can calibration models be transferred to sites without reference?** Yes for ${\mathrm{PM}}_{2.5}$ in both directions, with limited $R^{2}$ loss ($|\Delta R^{2}|$≤0.18) when the source model is applied unchanged (Section 4.4). The ${\mathrm{NO}}_{2}$ ML models fall to $R^{2}$≤0.18 at the destination, and choosing the model with the destination reference does not raise it above 0.22 (Table 7). The linear ${\mathrm{NO}}_{2}$ calibration keeps $R^{2}=0.21$ and 0.25 at the destination, within 0.08 of a linear calibration fitted there, but below the 0.33 and 0.37 of the best local calibration (Section 3.1 and Section 3.5). CO and O_3_ do not transfer, and the MQ131 gives no usable O_3_ signal even at its own site. For CO, the dependence on the unit-specific calibration of an individual mid-cost electrochemical sensor is the proposed explanation, but not the only possible one (Section 4.4).

**Practical recommendations for deployment:**

- **${\mathrm{PM}}_{2.5}$ (SPS30):** the strongest case in this study. Cross-site transfer is viable, and the calibration keeps describing the test data.
- **CO (GM-702B):** indicative monitoring with ML is feasible against an on-site factory-calibrated mid-cost electrochemical sensor, with seasonal retraining (Section 3.4).
- **${\mathrm{NO}}_{2}$ (GM-102B):** low to moderate performance ($R^{2}=0.23$ to 0.37) with ML selected without the test period. It outperforms the mid-cost electrochemical sensor on the test months, but both calibrations stop describing the test data, and a co-location at each site is required.
- **O_3_:** do not deploy the MQ131 for quantitative monitoring; explore the use of an electrochemical or ultraviolet (UV) absorption sensor.
- **Calibration method:** fit the linear OLS with temperature and relative humidity first. For ${\mathrm{PM}}_{2.5}$ it is sufficient and transfers between sites. Use ML where the OLS fails, as for the CO sensor, and treat its gain for ${\mathrm{NO}}_{2}$ as site-specific.
- **Network design:** calibrate at government reference sites and transfer ${\mathrm{PM}}_{2.5}$ models unchanged. A temporary co-location at the destination recovers part of the loss for models with an OLS input. Multi-site validation beyond two nodes is still needed before recommending universal transfer.

**Future work** extends in four directions. First, a companion study, using the three indoor-outdoor sensor pairs (Nodes 1↔2, 3↔4, 6↔7), will assess how reliably outdoor ${\mathrm{PM}}_{2.5}$, ${\mathrm{NO}}_{2}$ and CO can be inferred from indoor measurements across different building typologies. Second, the multi-tier dataset, currently being extended to a 12-month cycle, will be released as an open-access repository: the first public field record of the GM-702B and GM-102B alongside government-grade references. Third, the seven-node deployment offers a setting to test whether calibrated low-cost sensors can resolve the neighborhood-scale microclimates motivated in Section 1.1, and the hardware, software, and operational experience here can feed a practical do-it-yourself playbook for indoor and outdoor air quality hubs. Fourth, future O_3_ calibration may benefit from jointly modeling ${\mathrm{NO}}_{2}$ and O_3_> to capture the photochemical coupling that drives ozone variability at urban roadsides, rather than treating O_3_ as an independent temporal signal.

## Figures and Tables

**Table 1:** Deployment characteristics of the two analyzed nodes. The co-located reference is the high-cost LAQN government station next to each node (${\mathrm{NO}}_{2}$ by chemiluminescence, ${\mathrm{PM}}_{2.5}$ by Met One BAM); both nodes deploy outdoor at urban traffic roadsides.

Node	Site	Co-Located Ref.	Coordinates
3	QMUL	TH2 (LAQN)	51.522530^∘^ N, 0.042155^∘^ W
5	KEMP	TH7 (LAQN)	51.509477^∘^ N, 0.050541^∘^ W

**Table 2:** Three-tier sensor architecture and approximate costs. T: temperature; RH: relative humidity.

Sensor	Target
**Low-cost (1×): $7–$42/sensor**	
SHT45	T, RH
SGP40	VOC
GM-702B	CO
GM-102B	${\mathrm{NO}}_{2}$
MQ131	O_3_
SPS30	${\mathrm{PM}}_{2.5}$, ${\mathrm{PM}}_{10}$
**Mid-cost (5–7×): $200–270/sensor**	
Alphasense CO-B4	CO
Alphasense ${\mathrm{NO}}_{2}$-B43F	${\mathrm{NO}}_{2}$
Alphasense OX-B431	O_3_
**High-cost (700–1000×): $27,000/station**	
LAQN chemiluminescence	${\mathrm{NO}}_{2}$
Met One BAM	${\mathrm{PM}}_{2.5}$

Approximate 2026 prices (1 GBP = 1.35 USD, April 2026), subject to annual variability. The high-cost figure is the estimate of Section 1.1 for a complete reference station hosting two or more sensors; a per-sensor cost breakdown is not publicly available.

**Table 3:** Data coverage for Nodes 3 and 5 and their co-located LAQN reference stations.

Source	Records	${\mathrm{NO}}_{2}$ Miss.	${\mathrm{PM}}_{2.5}$ Miss.
Node 3	525,458	<0.01%	<0.01%
Node 5	515,962	2.63%	7.66%
TH2	29,282	6.09%	7.47%
TH7	29,330	0.12%	14.56%

**Table 4:** OLS calibration regressions per pollutant. All regressions include temperature (T) and relative humidity (RH) as covariates ($\beta_{2}$, $\beta_{3}$) and are fitted to 15-min averages, the time resolution of the reference data.

Pollutant	Signal ($\beta_{1}$)	Reference or Mid-Cost Sensor
CO	ratio_CO (GM-702B)	Alphasense CO-B4 (mid-cost)
${\mathrm{NO}}_{2}$ (low-cost)	ratio_${\mathrm{NO}}_{2}$ (GM-102B)	TH2/TH7 (high-cost)
${\mathrm{NO}}_{2}$ (mid-cost)	Alphasense post-AAN803	TH2/TH7 (high-cost)
O_3_	ratio_O_3_ (MQ131)	Alphasense OX-B431 (mid-cost)
${\mathrm{PM}}_{2.5}$	SPS30 raw	TH2/TH7 (high-cost)

**Table 7:** Cross-node transfer of the validation-selected models in the three scenarios of Section 2.7, scored on the destination test period. LGBM: LightGBM; XGB: XGBoost. $R_{s}^{2}$ and $R_{d}^{2}$: test $R^{2}$ at the source and destination nodes; $\Delta R^{2}$ = $R_{d}^{2}$−$R_{s}^{2}$; Dest.-selected: destination-selected transfer (Section 2.7).

Gas	Dir	Selected Model	$R_{s}^{2}$	Direct $R_{d}^{2}$ ($\Delta R^{2}$)	OLS-Refitted $R_{d}^{2}$ ($\Delta R^{2}$)	Dest.-Selected Model	Dest.-Selected $R_{d}^{2}$
${\mathrm{PM}}_{2.5}$	N3→N5	XGB-B	0.74	0.56 (−0.18)	0.61 (−0.13)	XGB-B	0.61
${\mathrm{PM}}_{2.5}$	N5→N3	RF-C	0.58	0.66 (+0.08)	0.66 (+0.08)	XGB-B	0.72
${\mathrm{NO}}_{2}$	N3→N5	LGBM-C	0.23	0.04 (−0.19)	0.02 (−0.21)	RF-B	0.20
${\mathrm{NO}}_{2}$	N5→N3	XGB-B	0.37	0.18 (−0.19)	0.22 (−0.16)	XGB-B	0.22
CO	N3→N5	XGB-B	0.79	0.02 (−0.77)	0.21 (−0.58)	DNN-B	0.24
CO	N5→N3	RF-A	0.58	0.28 (−0.30)	n/a ^1^	RF-A	0.28
O_3_	N3→N5	RF-B	0.00	−0.56 (−0.56)	−0.57 (−0.57)	LSTM-A	−0.01
O_3_	N5→N3	LSTM-A	−2.10	−0.70 (+1.40)	n/a ^1^	LSTM-B	0.17

^1^ Not applicable: the selected model uses Config A, which has no OLS input to refit.

## Data Availability

The raw node data of this study are openly available in the qm-airquality-network-data repository (https://github.com/AndresMercad0/qm-airquality-network-data (accessed on 6 October 2026)), and the node firmware and hardware documentation in the qm-airquality-network repository (https://github.com/AndresMercad0/qm-airquality-network (accessed on 6 October 2026)). The calibration pipeline, the model-selection, ablation and transfer scripts of this revision, and the configuration files needed to reproduce the tables and figures are available in the qm-airquality-network-calibration repository (https://github.com/AndresMercad0/qm-airquality-network-calibration (accessed on 6 October 2026)), archived at https://doi.org/10.5281/zenodo.22850320. That repository also holds the LAQN reference records as downloaded on 15 February 2026, which are the values used in this study. Current LAQN data are available from the London Air Quality Network (https://www.londonair.org.uk (accessed on 6 October 2026)).

## Appendix A. Input Features of the Machine Learning Models

All input features of the three configurations of Section 2.6 are prepared in the same three steps. First, the 30 s samples that pass the quality control of Section 2.2 are averaged in 15-min intervals, the time resolution of the reference data. Second, no missing value is imputed: a 15-min observation is used for a given target and configuration only if all its features and the value of its reference or mid-cost sensor are available, which leaves the numbers of observations given in Table A3. Third, the tree-based models (RF, LightGBM and XGBoost) receive the features unscaled, whereas for the DNN and the LSTM each feature is standardized with the mean and standard deviation of the training data. Table A1 gives the source, unit and transformation of each feature.

Two questions decide whether these features could be used in real time: whether they need reference data, and whether they need samples recorded after the prediction. They need no reference data, because the only feature built with it, the OLS prediction of Configs B and C, applies coefficients fitted on the training data and then kept fixed. They do use later samples, in two steps of the offline processing. The Hampel filter of Section 2.2 compares each sample with the 20 samples on either side of it, so a real-time version would deliver each prediction 10 min late. The OLS prediction of the gas targets uses the $R_{0}$ of Section 2.3, estimated each day from the samples of 00:00 to 04:59 UTC of that day and of the 13 days before it. A prediction made in those hours thus uses later samples of the same night, and a real-time version would use the $R_{0}$ of the previous day, which differs by a median of 0.4% to 1.5% across days. Real-time use is therefore possible, at the cost of these two changes.

**Table A1:** Input features by configuration. T: temperature; RH: relative humidity.

Feature	Source	Unit	Transformation	Configs
${\mathrm{NO}}_{2}$ raw signal	GM-102B (Grove Multichannel v2, 10-bit ADC)	counts (0–1023)	none	A, B (gas targets)
CO raw signal	GM-702B (Grove Multichannel v2, 10-bit ADC)	counts (0–1023)	none	A, B (gas targets)
O_3_ signal	MQ131 (FC-22 board)	V	none	A, B (gas targets)
Temperature	SHT45	^∘^C	none	A, B, C
Relative humidity	SHT45	%	none	A, B, C
Absolute humidity	derived from T and RH	g/m^3^	Magnus formula	A, B, C
VOC raw signal	SGP40	ticks	humidity-compensated raw signal of the sensor (not the VOC index)	A, B
${\mathrm{PM}}_{2.5}$	SPS30	μg/m^3^	none	A, B (gas targets and ${\mathrm{PM}}_{2.5}$ target)
${\mathrm{PM}}_{10}$	SPS30	μg/m^3^	none	A, B (${\mathrm{PM}}_{2.5}$ target only)
Hour of day	time stamp (UTC)	-	sine and cosine of the hour of day (period 24 h)	A, B, C
Day of week	time stamp (UTC)	-	sine and cosine of the day of week (period 7 d)	A, B, C
OLS prediction	Equation (6) fitted on the training data	μg/m^3^	linear in signal, T and RH (signal = $R_{S}$/$R_{0}$ ratio for the MOS gases, SPS30 ${\mathrm{PM}}_{2.5}$ for ${\mathrm{PM}}_{2.5}$, ${\mathrm{NO}}_{2}$-B43F concentration for the mid-cost target)	B, C
Mid-cost ${\mathrm{NO}}_{2}$ (mid-cost target only)	Alphasense ${\mathrm{NO}}_{2}$-B43F after AAN-803-05	μg/m^3^	Algorithm 1 of [25]	A, B

The mid-cost ${\mathrm{NO}}_{2}$ target uses its own concentration as the signal, with T, RH, absolute humidity and the temporal features (8 features in Config A and 9 in Config B); the low-cost signals are not used.

## Appendix B. Hyperparameter Search, Model Selection and Software

**Search spaces.** Table A2 lists the hyperparameter space of each model family. RandomizedSearchCV samples configurations at random from the space (seed 42), scores each one by the mean negative RMSE over the five folds of a TimeSeriesSplit on the training data, and refits the best configuration on all training data. The search uses the OLS input of Configs B and C fitted once on all training data, without refitting it in each fold. Refitting the OLS in each fold was tested for the seven validation-selected tree models that use it. Three keep their hyperparameters, and the test $R^{2}$ of the other four changes by −0.24 to +0.14, with no consistent direction and no change in the comparison with the train-only OLS. Tree ensembles use the library defaults for every parameter not listed.

**Architectures and training.** The DNN is a multi-layer perceptron with two hidden layers (ReLU activation and dropout after each) and a linear output, trained with Adam and the mean squared error loss. The LSTM has one recurrent layer of lstm_units, a fully connected layer of fc_dim (ReLU) and a linear output. It reads windows of 96 consecutive 15-min observations (24 h) and predicts the last observation of each window. Windows are built only within segments without gaps longer than 30 min, both during the search and in the final fit, and the search scores the LSTM only on the observations for which such a window exists. The other observations are ignored, not counted as errors. The DNN and the LSTM standardize their inputs with statistics from the training part of each fold only. Both networks hold out the last 15% of their training observations as an internal validation set and stop early after 20 epochs without improvement, restoring the best weights. After the search, LightGBM is refitted with early stopping (50 rounds) on the same last-15% block. Every model is seeded with 42 (scikit-learn random_state, torch.manual_seed before each fit). GPU kernels can introduce small non-determinism in the DNN and LSTM. The LSTM values of Figure 9 and Table 5 come from this search.

**Table A2:** Hyperparameter search spaces and number of sampled configurations per combination. LGBM: LightGBM; XGB: XGBoost.

Model	Space	Sampled
RF	n_estimators 100, 200, 500, 1000; max_depth 5, 10, 20, 30, none; min_samples_split 2, 5, 10; min_samples_leaf 1, 2, 4; max_features sqrt, log2, 0.5, 0.8	100
LGBM	n_estimators 100, 200, 500, 1000; max_depth 3, 5, 7, 10, none; learning_rate 0.01, 0.05, 0.1, 0.2; num_leaves 15, 31, 63, 127; min_child_samples 5, 10, 20, 50; subsample and colsample_bytree 0.6, 0.8, 1.0; reg_alpha and reg_lambda 0, 0.1, 1.0	100
XGB	n_estimators 100, 200, 500, 1000; max_depth 3, 5, 7, 10; learning_rate 0.01, 0.05, 0.1, 0.2; min_child_weight 1, 3, 5, 10; subsample and colsample_bytree 0.6, 0.8, 1.0; reg_alpha and reg_lambda 0, 0.1, 1.0	100
DNN	hidden_dim_1 32, 64, 128; hidden_dim_2 16, 32, 64; dropout 0.1, 0.2, 0.3; learning rate 1 × 10^−4^, 5 × 10^−4^, 1 × 10^−3^, 5 × 10^−3^; batch size 32, 64, 128; epochs 100, 200, 300	40
LSTM	lstm_units 32, 64, 128; fc_dim 16, 32, 64; dropout 0.1, 0.2, 0.3; learning rate 1 × 10^−4^, 5 × 10^−4^, 1 × 10^−3^; batch size 32, 64; epochs 100, 200	20

**Combinations without a result.** At Node 3, six of the 75 combinations produce no prediction: the LSTM for the low-cost ${\mathrm{NO}}_{2}$ and for the mid-cost ${\mathrm{NO}}_{2}$ in each of the three configurations. The LSTM reads windows of 96 consecutive valid observations (24 h), but the TH2 ${\mathrm{NO}}_{2}$ reference, against which both targets are trained, lacks the 00:45 and 01:00 UTC values on 293 of the 306 days of the study. The longest run of consecutive values in that series is therefore 94, and no window can be formed (Section 4.5).

**Effective observations per target.** Table A3 gives the number of training and test observations per target after removing the observations with a missing feature or reference value. The 29376 15-min observations per node run from 16 April 2025 00:00 to 15 February 2026 23:45 UTC. Config C keeps more observations for CO and O_3_ at both nodes and for ${\mathrm{NO}}_{2}$ at Node 5, because it does not use the raw signals of the other two gas sensors. An LSTM predicts only the observations preceded by 24 h of consecutive values (see above), so it predicts fewer observations than the other models of its configuration and none for the two ${\mathrm{NO}}_{2}$ targets at Node 3.

**Table A3:** Effective 15-min observations per target and node (train/test). Config B equals Config A except for ${\mathrm{NO}}_{2}$ at Node 3 (18,100/5174) and Node 5 (13,577/7255).

Target	Node 3, Config A	Node 3, Config C	Node 5, Config A	Node 5, Config C
${\mathrm{PM}}_{2.5}$	16,848/6144	16,848/6144	15,114/3701	15,114/3703
CO	17,409/6271	17,484/6328	13,391/7094	15,390/7836
${\mathrm{NO}}_{2}$ (low-cost)	18,181/5174	18,100/5177	13,668/7255	14,951/7971
${\mathrm{NO}}_{2}$ (mid-cost)	16,813/5185	16,813/5185	15,528/7829	15,528/7829
O_3_	17,410/6271	17,485/6324	13,391/7094	14,643/7836

**Ablation inputs.** The models of Table 6 are Random Forests trained with the search of Table A2 (100 sampled configurations, seed 42). For each target and node, the five reduced models and Config A were trained and tested on the same observations, so the six values in each line of Table 6 are directly comparable. Set 1 contains temperature, relative humidity, absolute humidity, and the sine and cosine of hour and weekday (7 features). Set 2 adds the SGP40 raw signal and the SPS30 ${\mathrm{PM}}_{2.5}$ to set 1 (9 features). For the ${\mathrm{PM}}_{2.5}$ target, set 2 adds the SGP40 only (8 features). Set 3 contains only the raw channel of the target sensor: the ADC counts of the GM-102B or GM-702B, or the SPS30 ${\mathrm{PM}}_{2.5}$ (1 feature). Set 4 contains the three MOS channels (3 features) or, for the ${\mathrm{PM}}_{2.5}$ target, the SPS30 ${\mathrm{PM}}_{2.5}$ and ${\mathrm{PM}}_{10}$ readings (2 features). Sets 2 and 4 together form Config A. Set 5 is Config A without the raw channel of the target sensor (11 features). For the ${\mathrm{PM}}_{2.5}$ target it removes both SPS30 readings and therefore equals set 2.

**Model selection.** Table A4 lists, for each target and node, the model selected by the rule of Section 2.6 (lowest validation RMSE), the model selected on the common validation observations (Section 2.6), the model that a parsimony rule would select instead, and the highest test $R^{2}$ among the 15 combinations. The parsimony rule takes the simplest model among those within 2% of the lowest validation RMSE, in the order RF, then XGBoost and LightGBM, then DNN, then LSTM. The validation block ends with the last valid training observation, 31 October 2025 at Node 3 and 11 October at Node 5. Depending on the target and the configuration, it starts between 31 August and 14 September at Node 3 and between 6 and 19 August at Node 5. Each combination was refitted on the training observations preceding the validation block, with the hyperparameters that the search of Table A2 had selected for it. The DNN and the LSTM were refitted with three seeds (42, 43, 44) and ranked by their mean validation RMSE. The two rules disagree in three cases, all at Node 3 (${\mathrm{PM}}_{2.5}$, ${\mathrm{NO}}_{2}$ and the mid-cost ${\mathrm{NO}}_{2}$), where the validation RMSE of the simpler model is 0.3 to 2.0% higher. Scoring the stored models again on their own test observations reproduces the test $R^{2}$ of the master table within 0.0003.

**Table A4:** Selected model per target and node under the two pre-specified rules, the model selected when all combinations are scored on their common validation observations, and the highest test $R^{2}$ among the 15 combinations (test $R^{2}$ in brackets; validation RMSE in μg/m^3^; LGBM: LightGBM; XGB: XGBoost).

Target	Node	Lowest Validation RMSE	Val. RMSE	Common Observations	Parsimony Rule	Highest Test $R^{2}$
${\mathrm{PM}}_{2.5}$	3	XGB-B (0.740)	2.82	XGB-B (0.740)	RF-B (0.761)	DNN-C (0.769)
${\mathrm{PM}}_{2.5}$	5	RF-C (0.580)	2.44	RF-C (0.580)	RF-C (0.580)	XGB-B (0.619)
CO	3	XGB-B (0.792)	49.0	XGB-B (0.792)	XGB-B (0.792)	LSTM-B (0.834)
CO	5	RF-A (0.577)	57.2	RF-A (0.577)	RF-A (0.577)	RF-A (0.577)
${\mathrm{NO}}_{2}$ (low-cost)	3	LGBM-C (0.228)	8.67	LGBM-C (0.228)	RF-C (0.540)	DNN-A (0.579)
${\mathrm{NO}}_{2}$ (low-cost)	5	XGB-B (0.373)	7.34	LGBM-A (0.360)	XGB-B (0.373)	LSTM-B (0.548)
${\mathrm{NO}}_{2}$ (mid-cost)	3	LGBM-B (−0.404)	9.88	LGBM-B (−0.404)	RF-B (0.135)	DNN-B (0.242)
${\mathrm{NO}}_{2}$ (mid-cost)	5	LSTM-C (−0.392)	6.38	XGB-B (−0.222)	LSTM-C (−0.392)	RF-A (−0.062)
O_3_	3	RF-B (−0.003)	17.4	RF-B (−0.003)	RF-B (−0.003)	LSTM-A (0.139)
O_3_	5	LSTM-A (−2.103)	17.3	LSTM-A (−2.103)	LSTM-A (−2.103)	LSTM-B (−0.509)

**OLS versus the selected ML model on the validation block.** Table A5 compares, on the same validation block, the train-only OLS of Section 2.5 with the validation-selected ML model of each target. The OLS was refitted on the training observations preceding the block and scored on the observations of the block for the selected configuration. The ML model has the lower validation RMSE in eight of the ten cases. The OLS has it for the mid-cost ${\mathrm{NO}}_{2}$ sensor at Node 3 and for ${\mathrm{PM}}_{2.5}$ at Node 5. Figure 12 reports, for each tier at Node 3, the method selected by this comparison. On the test period the OLS exceeds the selected ML model for ${\mathrm{PM}}_{2.5}$ and ${\mathrm{NO}}_{2}$ at Node 3 (Section 3.1). The validation block ends in October and does not anticipate that difference. For the two LSTM models the ML value refers to the observations of the block with a complete input window.

**Table A5:** Validation RMSE (μg/m^3^) of the train-only OLS and of the validation-selected ML model on the validation block of Section 2.6, and the method with the lower value. LGBM: LightGBM; XGB: XGBoost.

Target	Node	ML Model	Val. RMSE ML	Val. RMSE OLS	Lower RMSE
${\mathrm{PM}}_{2.5}$	3	XGB-B	2.82	2.96	ML
${\mathrm{PM}}_{2.5}$	5	RF-C	2.44	2.38	OLS
CO	3	XGB-B	49.0	64.1	ML
CO	5	RF-A	57.2	123.9	ML
${\mathrm{NO}}_{2}$ (low-cost)	3	LGBM-C	8.67	9.03	ML
${\mathrm{NO}}_{2}$ (low-cost)	5	XGB-B	7.34	10.42	ML
${\mathrm{NO}}_{2}$ (mid-cost)	3	LGBM-B	9.88	9.71	OLS
${\mathrm{NO}}_{2}$ (mid-cost)	5	LSTM-C	6.38	8.20	ML
O_3_	3	RF-B	17.4	27.7	ML
O_3_	5	LSTM-A	17.3	34.2	ML

**Software and hardware.** The analyses of this revision ran with Python 3.12.3, scikit-learn 1.9.0, LightGBM 4.6.0, XGBoost 3.2.0, PyTorch 2.12.0 (CUDA 13), NumPy 2.4.6 and pandas 3.0.3. The machine is a workstation with an Intel Xeon E-2124G processor (4 cores), 64 GB of RAM and an NVIDIA GeForce RTX 4060 Ti (16 GB). The original hyperparameter searches ran on the same machine earlier in 2026. The model files of those searches record scikit-learn 1.8.0, and the versions of the other libraries were not recorded. The models of Table 6 and the searches with the OLS refitted in each fold used scikit-learn 1.8.0, the version of the original searches, so that their results are comparable with those of the published models. The code is available as stated in the Data Availability Statement.

## Appendix C. Mid-Cost Electrochemical Sensors

The raw voltages of the three mid-cost sensors are converted to concentrations with the manufacturer’s algorithm and the factory constants of each unit. Each sensor (CO-B4, ${\mathrm{NO}}_{2}$-B43F and OX-B431, Section 2.1) outputs a working-electrode (WE) and an auxiliary-electrode (AE) voltage. The conversion follows Algorithm 1 of AAN-803-05 [25], which the note suggests for these three sensor types, in four steps. First, the electronic zero of each electrode is subtracted from its voltage. Second, the AE voltage is scaled by the temperature-dependent factor n_T(T) tabulated in the note and subtracted from the WE voltage. Third, the corrected voltage is divided by the factory sensitivity of the unit to obtain ppb. Fourth, ppb is converted to μg/m^3^ at the measured temperature and standard pressure. The OX-B431 responds to O_3_ and ${\mathrm{NO}}_{2}$ together. Following the same note, its ${\mathrm{NO}}_{2}$ signal is computed from the concentration of the co-located ${\mathrm{NO}}_{2}$-B43F and the ${\mathrm{NO}}_{2}$ cross-sensitivity of the unit, and is subtracted before the third step. The constants are those supplied by the manufacturer with each unit (Table A6). The quality control of Section 2.2 is applied to the converted series.

**Independent validation.** Only the ${\mathrm{NO}}_{2}$-B43F could be validated independently, because the LAQN stations next to both nodes measure ${\mathrm{NO}}_{2}$ but not CO or O_3_ (Section 2.1). On the test observations of Section 3.1, the ${\mathrm{NO}}_{2}$ concentrations obtained with this conversion follow the LAQN reference with a Pearson r of 0.66 at Node 3 and 0.52 at Node 5. Their level is displaced, with a mean bias error of +8.5 and +6.5 μg/m^3^, and their $R^{2}$ is −0.31 and 0.05 (Figure 7 for Node 3). The CO-B4 and the OX-B431 could not be checked in the same way.

**Table A6:** Factory calibration constants of the six Alphasense units used in this study, from the calibration sheets supplied with each sensor.

Node	Sensor	Serial	Gain (mV/nA)	WE Sensitivity (nA/ppm)	Sensitivity (mV/ppb)	Electronic Zero WE/AE (mV)	${\mathrm{NO}}_{2}$ Cross-Sensitivity (nA/ppm)
3	CO-B4	162820163	0.8	515	0.412	354/346	-
3	${\mathrm{NO}}_{2}$-B43F	202802447	−0.73	−395	0.288	230/233	-
3	OX-B431	204801016	−0.73	−470	0.343	231/238	−644
5	CO-B4	162571941	0.8	561	0.449	342/341	-
5	${\mathrm{NO}}_{2}$-B43F	202571638	0.8	−378	−0.303	247/245	-
5	OX-B431	204571017	0.8	−354	−0.283	231/227	−584.12

Node 5 units were supplied under order 83358 of 1 November 2023. The sign of the gain and of the sensitivity follows the board configuration recorded on each sheet; the conversion uses the magnitude of the sensitivity. Table A7 lists the datasheet specifications of the three sensor types.

**Table A7:** Manufacturer specifications of the three electrochemical sensor types (tested with the Alphasense low-noise circuit) [36,37,38]. RH: relative humidity.

Specification	CO-B4	${\mathrm{NO}}_{2}$-B43F	OX-B431
Sensitivity (nA/ppm)	420 to 650	−200 to −650	−225 to −750
Response time t90 (s)	<30	<80	<80
Zero current at 20 ^∘^C (nA)	+30 to −250	−80 to +80	−80 to +80
Noise, ±2 SD (ppb equivalent)	4	15	15
Range, limit of performance warranty (ppm)	1000	20	20
Zero drift (ppb equivalent per year)	<±100	0 to 20	0 to 20
Sensitivity drift (% per year)	<10	−20 to −40	−20 to −40
Operating life (months until 50% of signal)	>36	>24	>24
Temperature range (^∘^C)	−30 to 50	−30 to 40	−30 to 40
Humidity range (% RH, continuous)	15 to 90	15 to 85	15 to 85

Noise and drift are laboratory figures of the manufacturer; the OX-B431 responds to O_3_ and ${\mathrm{NO}}_{2}$ together (datasheet title: oxidising gas sensor, ozone plus nitrogen dioxide), which is why the ${\mathrm{NO}}_{2}$ compensation of AAN-803-05 [25] is applied.

**Uncertainty of the mid-cost sensors.** The uncertainty of the factory-calibrated mid-cost CO and O_3_ sensors is known in this study only from the manufacturer’s specifications (Table A7). The specified noise (±2 standard deviations) is 4 ppb for the CO-B4 and 15 ppb for the OX-B431. The specified sensitivity drift is below 10% per year for the CO-B4 and a loss of 20 to 40% per year for the OX-B431. These are laboratory figures, and the factory constants of Table A6 were not re-determined during the deployment. The O_3_ values of the OX-B431 also depend on the ${\mathrm{NO}}_{2}$-B43F through the ${\mathrm{NO}}_{2}$ correction described above, so a level error of that sensor propagates to the O_3_ series. Any error of these mid-cost sensors is therefore included in the CO and O_3_ results of Section 3 (Section 4.5).
